# Genomic delineation and description of species and within-species lineages in the genus *Pantoea*

**DOI:** 10.3389/fmicb.2023.1254999

**Published:** 2023-11-09

**Authors:** Katherine C. Crosby, Mariah Rojas, Parul Sharma, Marcela A. Johnson, Reza Mazloom, Brian H. Kvitko, Theo H. M. Smits, Stephanus N. Venter, Teresa A. Coutinho, Lenwood S. Heath, Marike Palmer, Boris A. Vinatzer

**Affiliations:** ^1^School of Plant and Environmental Sciences, Virginia Tech, Blacksburg, VA, United States; ^2^Graduate Program in Genetics, Bioinformatics, and Computational Biology, Virginia Tech, Blacksburg, VA, United States; ^3^Department of Computer Science, Virginia Tech, Blacksburg, VA, United States; ^4^Department of Plant Pathology, University of Georgia, Athens, GA, United States; ^5^Environmental Genomics and System Biology Research Group, Institute of Natural Resource Sciences, Zurich University of Applied Sciences, Wädenswil, Switzerland; ^6^Department of Biochemistry, Genetics and Microbiology, Forestry and Agricultural Biotechnology Institute, University of Pretoria, Pretoria, South Africa; ^7^Centre for Microbial Ecology and Genomics, University of Pretoria, Pretoria, South Africa; ^8^School of Life Sciences, University of Nevada, Las Vegas, Las Vegas, NV, United States; ^9^Department of Microbiology, University of Manitoba, Winnipeg, MB, Canada

**Keywords:** *Pantoea*, taxonomy, genomics, reverse ecology, core genome, pangenome analysis

## Abstract

As the name of the genus *Pantoea* (“of all sorts and sources”) suggests, this genus includes bacteria with a wide range of provenances, including plants, animals, soils, components of the water cycle, and humans. Some members of the genus are pathogenic to plants, and some are suspected to be opportunistic human pathogens; while others are used as microbial pesticides or show promise in biotechnological applications. During its taxonomic history, the genus and its species have seen many revisions. However, evolutionary and comparative genomics studies have started to provide a solid foundation for a more stable taxonomy. To move further toward this goal, we have built a 2,509-gene core genome tree of 437 public genome sequences representing the currently known diversity of the genus *Pantoea*. Clades were evaluated for being evolutionarily and ecologically significant by determining bootstrap support, gene content differences, and recent recombination events. These results were then integrated with genome metadata, published literature, descriptions of named species with standing in nomenclature, and circumscriptions of yet-unnamed species clusters, 15 of which we assigned names under the nascent SeqCode. Finally, genome-based circumscriptions and descriptions of each species and each significant genetic lineage within species were uploaded to the LINbase Web server so that newly sequenced genomes of isolates belonging to any of these groups could be precisely and accurately identified.

## Introduction

The goal of taxonomy is to develop an organized system of named groups (taxa) of described organisms that is conducive to meaningful identification of those organisms and permits clear communication about them (Baron, 1996). From medical, veterinary, and plant pathology perspectives, it is also important that the identification of an unknown as a member of a taxon enables inference of the pathogenic potential of the organism. In fact, identification plays a critical role in the choice of medical treatment and determination of contagion risk in the case of disease diagnostics, decision-making on disease control and management in the case of disease outbreaks, or in decision-making on biosafety levels in microbiology research (Bayot and King, 2022). It also plays a key role in environmental applications when assessing risks and deciding on regulatory actions concerning the issuance of a field permit or approval for the commercialization of a microbial product (OECD, 2003; Chandler et al., 2011). Furthermore, the name of any taxon to which an organism belongs should not raise any doubts about which group the name refers to.

The current evolutionary, rank-based, hierarchical taxonomy of prokaryotes from the phylum level to the species/subspecies level and the established rules of nomenclature provide a framework that generally allows us to reach the described goal. In particular, species names are associated with type strains (Parker et al., 2019), or more recently, type genomes under the SeqCode (Hedlund et al., 2022; Whitman et al., 2022), to provide anchoring and stability of names. However, the evolutionary relationships among prokaryotes are impossible to establish based on phenotypes. Furthermore, sequencing one or a small number of marker genes, such as the 16S rRNA gene, is not always sufficient to correctly determine how bacteria are related to each other (Hauben et al., 1997). This is one of the reasons why several named species that were validly published in the past were later revised and assigned to different genera or families, including species that are, at present, assigned to the genus *Pantoea* (Gavini et al., 1989; Palmer et al., 2018a), the focus of this article. With the advent of cost-effective next-generation sequencing, whole-genome sequencing and constructing accurate core genome phylogenies (where all genes present only once in each analyzed genome are used) have become possible. Moreover, genome sequence comparisons currently allow us to easily assign bacteria to species based on average nucleotide identity (ANI), whereby 95% ANI is the generally agreed-upon guideline for the delineation of bacteria belonging to the same species (Konstantinidis and Tiedje, 2005; Jain et al., 2018). In recent years, the Genome Taxonomy Database (GTDB) has leveraged core genome phylogenies and ANI to establish exclusively genome-based taxa from phylum to species rank (Parks et al., 2020) that reflect evolutionary relationships and have standardized breadths of diversity (Parks et al., 2018).

However, there is another problem inherent to prokaryotic taxonomy that cannot be solved when using the species and subspecies as the smallest units: Important phenotypes, for example, the host range of plant pathogens (Cai et al., 2011), can vary within species, are sometimes linked to the accessory genome, and, therefore, do not always align with phylogeny (Venter et al., 2022). In these cases, identification of an unknown at the species or subspecies level is not sufficient to lead to meaningful phenotype prediction.

Typically, organisms with a recent common ancestor are more likely to share phenotypes with each other than with more distant relatives because they have vertically inherited a higher number of shared genes and alleles. Moreover, frequent recombination within populations of closely related organisms that occupy the same ecological niche further increases the number of shared genes and alleles among the members of these populations (Arevalo et al., 2019; Palmer et al., 2019). Therefore, identification of an unknown as a member of a group that has a very recent common ancestor can be expected to provide a more accurate prediction of the phenotype of the unknown than identification at the species level. The Life Identification Number (LIN) approach takes advantage of this fact by continuing hierarchical rank-based taxonomy from the species rank toward the strain level using a strictly ANI-based approach (Marakeby et al., 2014; Vinatzer et al., 2017). LINs consist of a series of positions, each representing a different ANI threshold, with ANI thresholds increasing from the left to the right. Any group of related genomes can be easily named by the LIN prefix they share (Vinatzer et al., 2017). The more similar the genomes of the groups' members are and the further their LIN prefix extends to the right, the more phenotypes they can be expected to share. Therefore, as long as the reciprocal ANI of the members of a group can be accurately measured because it is not too low (~70%) and not too high (~99.95%; depending on genome quality), the group can be clearly circumscribed. We call such groups “LINgroups”.

The genus *Pantoea* is a taxon that has proven to be particularly difficult to classify based on phenotypes (Tambong, 2019) as it shares many characteristics with other genera such as *Erwinia, Enterobacter*, and *Pectobacterium* (Brady et al., 2007). With the advent of DNA analysis tools, some species in the genera *Erwinia, Enterobacter*, and *Pectobacterium* were transferred to the genus *Pantoea* (Gavini et al., 1989; Mergaert et al., 1993; Brady et al., 2008, 2010). By the end of 2022, 19 names of species in the genus *Pantoea* were validly published as summarized on the LPSN website (Parte et al., 2020) and described in the Bergey's Manual chapter for *Pantoea* (Palmer and Coutinho, 2022), two named species were effectively published but the names could not be validly published under the International Code of Nomenclature of Prokaryotes; and an additional 29 species clusters were found to qualify as species in the GTDB (release 207) based on core genome phylogeny and ANI but have not been named (Parks et al., 2018, 2020, 2022).

The name of the genus *Pantoea* (“of all sorts and sources”) reflects that this genus includes bacteria that have been found in a wide range of environments, including plants, animals, soils, components of the water cycle, humans, and the built environment (Palmer and Coutinho, 2022). Mechan Llontop et al. (2021) found that bacteria in the genus are among the most common and efficient rain-borne colonizers of tomato leaves, suggesting that members of the genus frequently migrate between compartments of the water cycle and plants, as well as possibly other environments, without having adapted to a particular environment. However, some *Pantoea* species or subspecies have adapted and live in more restricted environments, for example, the insect-transmitted plant pathogen *P. stewartii* subsp. *stewartii* (Mergaert et al., 1993). For other species in the genus, it is not clear if all lineages within the same species have a similar pathogenic potential or if some lineages are pathogenic and others are not. Most notably, within *P. agglomerans*, Rezzonico et al. (2009) found isolates from humans, plants, and the environment. Some *P. agglomerans* strains appear to be opportunistic human pathogens, while others have been found to have biocontrol activity against plant pathogens (Rezzonico et al., 2009; Lorenzi et al., 2022). To date, it has not been possible to distinguish between strains with different phenotypes based on their genomes (Sulja et al., 2022). This is a challenge with practical implications since determining if a potentially beneficial *P. agglomerans* strain could at the same time present a risk to crops and even agricultural workers is a key question for which regulators need an answer when deciding on field permits and commercialization (Bonaterra et al., 2014). Another problem is that gene sequences and genome sequences sometimes have the wrong taxonomic assignment in NCBI, in particular at the species rank, at which up to 10% of genomes of *Pantoea* strains were found to be misidentified or mislabeled (Tambong, 2019), thus confusing NCBI users about the identity of their query sequences.

To contribute to the advancement of the taxonomy of the genus *Pantoea* and to facilitate the correct and precise identification of newly sequenced genomes, we compared 559 genome sequences of the genus. We computed pairwise ANI values between all genomes and built a highly resolved core genome tree of 437 representative genomes. We also determined which clades correspond to population clusters based on recent horizontal gene transfer events and analyzed differences in gene content between some of the genetic lineages that belong to the same species to help us determine if these lineages may occupy different ecological niches. We also compiled ecological and phenotypic information from the literature and from genome metadata, and we determined which clades in the core genome tree correspond to named species or yet-unnamed species clusters circumscribed in GTDB, 15 of which we named here under the SeqCode (SeqCode Registry List: seqco.de/r:xfaladud). We finally circumscribed all these genetic lineages as LINgroups in LINbase (Tian et al., 2020) and added the corresponding descriptions so that any newly sequenced *Pantoea* genome can be identified as a member of any of these groups.

## Methods

Genomes classified by GTDB release 207 (Parks and Hugenholtz, 2022) as *Pantoea* and with over 90% completeness were downloaded from the NCBI Assembly database on 4 October 2022, while taxonomy data and genome quality data were downloaded directly from GTDB. Associated metadata about sources of isolation were downloaded from NCBI's BioSample and BioProject databases on 5 October 2022. Additional genomes of strains isolated from precipitation that had been identified as *Pantoea* based on 16S rRNA sequencing (Failor et al., 2017) were also analyzed and submitted to NCBI under Project PRJNA445714. Genomes and metadata were then uploaded to LINbase, and ANI-based LINs were assigned as previously described (Tian et al., 2020).

When multiple genomes were over 99.95% identical to each other based on LINflow (Tian et al., 2021) analysis, only one genome representative for such a group was used for further analysis. These genomes were annotated using Bakta version 1.4.0 (Schwengers et al., 2021), and pangenome analysis was performed using the software PIRATE (Bayliss et al., 2019). Core genes were identified based on their presence in 95% of all genomes. Two core genome trees were constructed using (i) maximum likelihood (ML) with ultrafast bootstrapping in IQ-TREE (version 2.2.0.3) (Minh et al., 2020) and (ii) approximate ML with the generalized time-reversible model and SH-like branch support in FastTree version 2.1.11 (Price et al., 2010). Differences in gene content between clades were determined by creating a presence–absence matrix for all genomes for all gene families from the PIRATE output file, -PIRATE.gene_families.ordered.tsv, whereby presence was marked as 1 and absence as 0. This file was then queried in R (version 4.2.1) to determine the differences in gene content between clades within various *Pantoea* species.

The same genomes used for the pangenome and core genome analyses were also used as input for PopCOGenT (Arevalo et al., 2019) to determine population clusters using a reverse ecology approach. PopCOGenT detects recent gene flow events between genomes by searching for regions without SNPs. Two or more genomes that have more frequent and longer SNP-free regions are considered to belong to the same ecologically and genetically distinct population.

Finally, all named species found in the List of Prokaryotic Names with Standing in Nomenclature (Parte et al., 2020), unnamed species clusters in GTDB release 207 (Parks and Hugenholtz, 2022), and other clades identified in the core genome tree were circumscribed in LINbase, and descriptions from the Results section of this study were added to these circumscriptions.

## Results

### ANI clustering, phylogenomics, and population genomics lead to largely congruent results

A total of 550 genomes with over 90% completeness were found to be classified in GTDB release 207 (Parks and Hugenholtz, 2022) as members of the genus *Pantoea*. Nine genomes of strains isolated from precipitation and identified as members of the genus *Pantoea* (Failor et al., 2017) were also included. Supplementary Table 1 lists these 559 genomes together with their taxonomic lineages based on NCBI and GTDB, with their ANI-based LINs expressing their precise genomic similarity to each other, with metadata NCBI BioSample data, NCBI BioProject data, and research articles, and a variety of additional metadata from NCBI and GTDB. Genomes of *Pantoea* species that were later reclassified as members of the genera *Mixta* and *Tatumella* or that were found by GTDB to belong to other genera were not included in our analysis.

Based on their assigned LINs (see Supplementary Table 1), 559 putative *Pantoea* genomes were found to have at least 80% pairwise ANI values and to belong to 437 groups with pairwise ANI values of over 99.95%. Only one genome for each of these 437 groups was retained for further analysis to provide a more balanced representation of the genus *Pantoea* than the original 559 genomes.

At the 95% ANI species threshold (LIN position F), 49 LINgroups were identified. These species-level LINgroups comprised between 1 and 147 genomes each. Eighteen of the LINgroups corresponded to validly published species under the genus *Pantoea*, four to effectively published named *Pantoea* species, two to effectively published species outside of the genus, and another twenty-five were provisionally named in GTDB as species clusters and do not correspond to either effectively or validly published species (Table 1; Supplementary Table 3). Three GTDB species clusters were not considered here since they had representative genomes that were <90% complete and had been excluded from our analysis (Table 1). Within some of the species-level LINgroups, LINgroups at higher ANI levels stood out. Such groups are discussed further in the next section, and their corresponding LINs are reported in Supplementary Table 3.

**Table 1:** *Pantoea* species names and etymology from the LPSN^a^ or the new species names proposed here, the nomenclatural type from the LPSN^a^ or proposed here, the name/number of the corresponding GTDB^b^ species/cluster, the GTDB^b^ representative and NCBI^c^ accession number, and synonyms.

**Species name and etymology**	**Nomenclatural type(s) and NCBI^c^ accession**	**GTDB^b^ species name/cluster (if different)**	**GTDB representative genome and NCBI^c^ accession**	**Synonyms**
*Pantoea agglomerans* (Gavini et al., 1989) ag.glo'me.rans. L. inf. v. *agglomerare*, to wind on (as on a ball); L. part. adj. *agglomerans*, forming into a ball (referring to the occurrence of symplasmata bacteria in aggregates surrounded by a translucent sheath in anaerogenic strains)	DSM 3493^T^ (ATCC 27155^T^, CCUG 539^T^, CDC 1461-67^T^, CFBP 3845^T^, CIP 57.51^T^, ICPB 3435^T^, JCM 1236^T^, LMG 1286^T^, NBRC 102470^T^, and NCTC 9381^T^) (GCF_001598475.1)	N/A^e^	NBRC 102470^T^ (GCF_001598475.1)	Homotypic synonyms: *Enterobacter agglomerans* Beijerinck 1888, “*Bacillus agglomerans*”. Heterotypic synonyms: *Erwinia milletiae* (Kawakami and Yoshida 1920) Magrou 1937 (Skerman et al., 1980); *Erwinia herbicola* (Lohnis, 1911) (Dye, 1964) (Skerman et al., 1980); “*Bacillus milletiae*” (Kawakami and Yoshida, 1920), “*Pantoea pleuroti*” (Ma Y. et al., 2016)
*Pantoea gossypiicola* sp. nov. gos.sy.pi.i.co'la. N.L. neut. n. *Gossypium*, the scientific botanical genus name for cotton; L. fem. n. suff. -*cola*, inhabitant, dweller; N.L. fem. n. *gossypiicola*, inhabitant of *Gossypium*, referring to the isolation source of the strain for the designated nomenclatural type, from the leaves of cotton plants	B_8^Ts^ (GCF_008632075.1)	sp008632075	B_8^Ts^ (GCF_008632075.1)	None
“*Pantoea alfalfae*” (Yao et al., 2023) al.fal′fae. L. n. *alfalfae* of alfalfa (*Medicago sativa* L.), referring to the host plant from which the first strains were isolated	CQ10^T^	sp003512445	CQ10^T^ (GCF_009905795.1)	None
*Pantoea vagans* (Brady et al., 2009) va'gans. L. part. adj. *vagans*, roaming, referring to the wide distribution of the species	LMG 24199^T^ (BCC 105^T^, BD 765^T^, DSM 23078^T^, and R 21566^T^)	N/A^e^	LMG 24199^T^ (GCF_004792415.1)	None
*Pantoea eucalypti* (Brady et al., 2009) eu.ca.lyp'ti. N.L. gen. n. *eucalypti*, of Eucalyptus, referring to the host from which the first strains were isolated	LMG 24197^T^ (BCC 76^T^, BD 769^T^, DSM 23077^T^, and R 25678^T^)	N/A^e^	LMG 24197^T^ (GCF_009646115.1)	“*Pantoea hericii*” (Rong et al., 2016)
*Pantoea rara* sp. nov. ra'ra L. fem. adj. *rara*, sporadic or uncommon, referring to the sporadic isolation and recovery from disparate sources of members of this species	UBA5707^Ts^ (GCA_002419935.1)	sp002419935	WMus005 (GCF_013415305.1)	None
*Pantoea varia* sp. nov. va'ri.a. L. fem. adj. *varia*, varied, corresponding to the varying sources of isolation for members of this species	OV426^Ts^ (GCF_900115075.1)	sp900115075	OV426^Ts^ (GCF_900115075.1)	None
*Pantoea deleyi* (Brady et al., 2009) de.ley'i. N.L. gen.) n. *deleyi*, of De Ley, named for Jozef De Ley, who contributed to the formation of the genus *Pantoea*	R 31523^T^ (LMG 24200^T^, BCC 109^T^, BD 767^T^, and DSM 23079^T^)	N/A^e^	LMG 24200^T^ (GCF_006494415.1)	None
*Pantoea* sp003236715	N/A^e^	sp003236715	ARC607 (GCF_003236715.1)	None
*Pantoea anthophila* (Brady et al., 2009) an.tho'phi.la. Gr. neut. n. *anthos*, flower; Gr. masc. adj. *philos*, loving; N.L. fem. adj. *anthophila*, flower-loving, pertaining to the source of isolation of known strains	LMG 2558^T^ (BD 871^T^, DSM 23080^T^, and NCPPB 1682^T^)	N/A^e^	LMG 2558^T^ (GCF_006494375.1)	None
*Pantoea* sp009830035	N/A^e^	sp009830035	Eser (GCA_009830035.1)	None
*Pantoea conspicua* (Brady et al., 2010) con.spi'cu.a. L. fem. adj. *conspicua*, conspicuous, referring to the conspicuous separation from other strains within DNA hybridization group V	LMG 24534^T^ (CDC 3527^T^, CDC 3527-71^T^, BD 805^T^, and DSM 24241^T^)	N/A^e^	LMG 24534^T^ (GCF_002095315.1)	None
*Pantoea astica* sp. nov. as.ti'ca Gr. masc. adj. *astikós*, of a city; N.L. fem. adj. *astica*, of a city, referring to the recovery of this organism from New York City, NY, USA	UBA6564^Ts^ (GCA_002434205.1)	sp002434205	UBA6564^Ts^ (GCA_002434205.1)	None
*Pantoea ananatis* (Mergaert et al., 1993) N.L. masc. n. *Ananas*, generic name of the pineapple; N.L. gen. n. *ananatis*, of *Ananas*, of the pineapple	LMG 2665^T^ (ATCC 33244^T^, CFBP 3612^T^, CIP 105207^T^, DSM 17873^T^, ICPB ES175^T^, NCPPB 1846^T^, and PDDCC 1850^T^)	N/A^e^	LMG 2665^T^ (GCF_000710035.2)	Homotypic synonyms: *Erwinia ananatis* corrig. Serrano 1928 (Approved Lists 1980), *Erwinia ananas* Serrano, 1928 (Approved Lists 1980) [inaccurate spelling]. Heterotypic synonyms: “*Xanthomonas uredovorus*” Pon et al. 1954; *Erwinia uredovora* (Pon et al., 1954) Dye 1963 (Approved Lists 1980). Other spelling of name: *Pantoea ananas* (Serrano, 1928) Mergaert et al., 1993 [inaccurate spelling]
*Pantoea allii* (Brady et al., 2011) al'li.i. L. neut. n. *Allium*, the scientific generic name of the onion (*Allium* sp.); from L. neut. n. *allium*, garlic; L. gen. n. *allii*, of/from *Allium*, referring to the isolation of the first strains from *Allium cepa* L.	LMG 24248^T^ (BD 390^T^ and DSM 25133^T^)	N/A^e^	LMG 24248^T^ (GCF_002307475.1)	None
*Pantoea stewartii* (Mergaert et al., 1993) stew.art'i.i. N.L. gen. n. *stewartii*, of Stewart, named after F.C. Stewart, the plant pathologist who first described the wilt disease caused by *P. stewartii* subspecies *stewartii* on corn	ATCC 8199^T^ (CFBP 2349^T^, CFBP 3167^T^, CIP 104005^T^, DSM 30176^T^, ICMP 257^T^, ICPB SS11^T^, IMET 11187^T^, LMG 2715^T^, NCPPB 2295^T^, NRRL B-794^T^, and CCUG 26359^T^)	N/A^e^	CCUG 26359^T^ (GCF_008801695.1)	Homotypic synonyms: “*Pseudomonas stewarti*” Smith, 1898, *Erwinia stewartii* (Smith, 1898) Dye 1963 (Approved Lists 1980)
*Pantoea bituminis* sp. nov. bi.tu.mi'nis N.L. gen. n. *bituminis*, of bitumen, asphalt, where this organism was isolated from	EnvD^Ts^ (GCF_018842675.1)	sp018842675	EnvD^Ts^ (GCF_018842675.1)	None
*Pantoea* “mediterraneensis_A”	Marseille-Q2057^T^	mediterraneensis_A	Marseille-Q2057^T^ (GCF_014946725.1)	This GTDB species cluster corresponds to the effectively published species “*Mixta mediterraneensis”* (Boxberger et al., 2021)
*Pantoea haifensis* sp. nov. hai.fen'sis N.L. fem. adj. *haifensis*, of Haifa, Israel, where this organism was isolated from	EnvH^Ts^ (GCA_018842655.1)	sp018842655	EnvH^Ts^ (GCA_018842655.1)	None
*Pantoea eucrina* (Brady et al., 2010) eu.cri'na. N.L. fem. adj. *eucrina*; from Gr. masc./fem. adj. eukrinês, well-separated, referring to the clear separation of the strains from other species within the genus	LMG 5346^T^ (BD 872^T^, CDC 1741^T^, CDC 1741-71^T^, and DSM 24231^T^)	N/A^e^	LMG 5346^T^ (GCF_002095385.1)	None
*Pantoea borealis* sp. nov. bo.re.a'lis. L. fem. adj. *borealis*, pertaining to the North, referring to the Northern hemisphere where this organism was recovered from	UBA6694^Ts^ (GCA_002454735.1)	eucrina_A	Russ (GCF_001691555.1)	None
*Pantoea soli* sp. nov. so'li. L. gen. n. *soli*, of soil, referring to the isolation source of the strain from which the type was derived	DSM 32899^Ts^	dispersa_B	DSM 32899^Ts^ (GCF_007833795.1)	None
*Pantoea wallisii* (Brady et al., 2012) wal.li'si.i. N.L. gen. n. *wallisii*, of Wallis, named after F. M. Wallis for his contribution to the field of phytobacteriology in South Africa	LMG 26277^T^ (BCC 682^T^, BD 946^T^, and DSM 26586^T^)	N/A^e^	LMG 26277^T^ (GCF_002095485.1)	None
*Pantoea symbiotica* sp. nov. sym.bio'ti.ca. Gr. fem. adj. *symbios*, a companion, partner, N.L. fem. adj. *symbiotica*, living together, referring to the isolation and recovery of genomes of this species from other organisms	YR512^Ts^ (GCF_900114175.1)	ludwigii_B	EnVs6 (CEFO00000000)	None
“*Pantoea endophytica*” (Gao et al., 2019) en.do.phy'ti.ca. Gr. pref. *endo-*, within; Gr. neut. n. *phyton*, plant; L. fem. adj. suff. *-ica*, used with the sense of belonging to; N.L. fem. adj. *endophytica*, within plant, endophytic	596^T^ (CGMCC 1.15280^T^ and DSM 100785^T^)	endophytica	596^T^ (GCF_002858935.1)	A homonym exists for “*P. endophytica”* (Gao et al., 2019): “*Pantoea endophytica”* (Ripka, 2005). Ripka neither officially proposed the name nor properly cited the name's authority.
*Pantoea* “ludwigii_A”	N/A^e^	ludwigii_A	EnVs2 (GCA_900068845.1)	None
“*Pantoea nemavictus”* (Dirksen et al., 2020)	BIGb0393^T^ (GCF_013450275.1)	sp001187905	RIT-PI-b (GCF_001187905.1)	None
*Pantoea floridensis* sp. nov. flo.ri.den'sis N.L. fem. adj. *floridensis*, of or from Florida, referring to where this organism was isolated from	JKS000234^Ts^ (GCA_900215435.1)	sp900215435	JKS000234^Ts^ (GCA_900215435.1)	None
*Pantoea communis* sp. nov. com.mu'nis. L. fem. adj. *communis*, common or widespread, referring to the broad global distribution of members of this species	Al-1710^Ts^ (GCF_011752685.1)	cancerogena_A	M004 (JRUP00000000)	None
*Pantoea* “rwandensis_B”	N/A^e^	rwandensis_B	ND04 (GCF_000759475.1)	None
*Pantoea* sp016625195	N/A^e^	sp016625195	S63 (GCF_016625195.1)	None
*Pantoea rodasii* (Brady et al., 2012) ro.da'si.i. N.L. gen. n. *rodasii*, of Rodas, named after Carlos Rodas for his contribution to forest pathology in Colombia	LMG 26273^T^ (BD 943^T^, BCC 581^T^, and DSM 26611^T^)	N/A^e^	LMG 26273^T^ (GCF_002811195.1)	None
*Pantoea* “vagans_C”	N/A^e^	vagans_C	ND02 (GCF_001506165.1)	None
*Pantoea formicae* sp. nov. for.mi'ca.e. L. gen. n. *formicae*, of an ant, referring to the ants propagating the fungal gardens from which the isolate used to determine the nomenclatural type was isolated	Acro-805^Ts^	sp011752625	Acro-805^Ts^ (GCF_011752625.1)	None
*Pantoea rwandensis* (Brady et al., 2012) rwan.den'sis. N.L. masc./fem. adj. *rwandensis*, of or belonging to Rwanda, referring to the country of isolation	LMG 26275^T^ (BCC 571^T^, BD 944^T^, and DSM 26585^T^)	N/A^e^	LMG 26275^T^ (GCF_002095475.1)	None
*Pantoea cypripedii* (Brady et al., 2010) cyp.ri.pe'di.i. N.L. neut. n. *Cypripedium*, botanical name for a genus of orchid; N.L. gen. n. *cypripedii*, of cypripedium orchids	LMG 2657^T^ (ATCC 29267^T^, CFBP 3613^T^, CIP 105195^T^, DSM 3873^T^, NCPPB 3004^T^, and PDDCC 1591^T^)	N/A^e^	LMG 2657^T^ (GCF_002095535.1)	Homotypic synonyms: “*Bacillus cypripedii*” Hori 1911 [basonym of name in Approved Lists], *Erwinia cypripedii* (Hori, 1911) Bergey et al. 1923 (Approved Lists 1980), *Pectobacterium cypripedii* (Hori, 1911) Brenner et al. 1973 (Approved Lists 1980)
*Pantoea* “cypripedii_A”	N/A^e^	cypripedii_A	NE1 (GCF_011395035.1)	None
*Pantoea* sp000468095	N/A^e^	sp000468095	AS-PWVM4 (GCF_000468095.1)	None
*Pantoea* sp000175935	N/A^e^	sp000175935	At-9b (GCF_000175935.2)	None
*Pantoea multigeneris* sp. nov. mul.ti.ge.ne'ris L. gen. n. *multigeneris*, of different kinds, varied, referring to the varied sources of isolation of this organism	Acro-835^Ts^ (GCF_011752615.1)	sp000295955	A4^Ts^ (GCF_000295955.2)	None
*Pantoea septica* (Brady et al., 2010) sep'ti.ca. L. fem. adj. *septica*, producing a putrefaction, putrefying, septic; from Gr. masc. adj. *sêptikos*, putrefying, decaying or septic, referring to the septicaemia outbreak associated with these strains	LMG 5345^T^ (BD 874^T^, CDC 3123^T^, CDC 3123-70^T^, and DSM 24604^T^)	N/A^e^	LMG 5345^T^ (GCF_002095575.1)	None
*Pantoea alvi* sp. nov. al'vi. L. gen. n. *alvi*, of the bowels, referring to the putative association of this species with the gastrointestinal tract of humans	PSNIH6^Ts^	sp002920175	PSNIH6^Ts^ (GCF_002920175.1)	None
*Pantoea piersonii* (Singh et al., 2019) Palmer and Coutinho, 2022 pier'so.ni.i. N.L. gen. n. *piersonii*, referring to Duane Pierson, an accomplished American space microbiologist	DSM 108198^T^ (IIIF1SW–P2 and NRRL B-65522)	N/A^e^	IIIF1SW-P2^T^ (GCF_003612015.1)	Homotypic synonyms: “*Pantoea piersonii*” (Singh et al., 2019) Soutar and Stavrinides, 2022; *Kalamiella piersonii* Singh et al. 2019
“*Pantoea latae”* (no etymology) (Lata et al., 2017)	AS1^T^	latae	AS1^T^ (GCF_002077695.1)	None
*Pantoea deserta* sp. nov. de.ser'ta L. fem. adj. *deserta*, deserted or alone, referring to the species having a sole representative at the time of description	RIT388^Ts^	sp003813865	RIT388^Ts^ (GCF_003813865.1)	None
*Pantoea* “mediterraneensis” me.di.ter.ra.ne.en'sis. N.L. masc./fem. adj. *mediterraneensis*, of Medi-terraneum, the Latin name of the Mediterranean Sea by which Marseille is located, and where strain P5165 was isolated	Marseille- P5165^T^	mediterraneensis	Marseille-P5165^T^ (GCF_900604315.1)	Basonym: “*Erwinia mediterraneensis*” (Ndiaye et al., 2019)
*Pantoea superficialis* sp. nov. su.per.fi.ci.a'lis L. fem. adj. *superficialis*, of or pertaining to a surface	UBA648^Ts^ (GCA_002299595.1)	sp002359495	UBA2655^Ts^ (DENB00000000)	None
*Pantoea* sp001485295	N/A^e^	sp001485295	PS-ISGKf71^f^ (GCA_001485295.1)	None
*Candidatus* Pantoea persica (Kashkouli et al., 2021) per'si.ca. L. fem. adj. *persica*, Persian (Parte et al., 2020)	Rafsanjan1^T^	persica	Kerman3^f^ (GCA_013753875.1)	None
*Pantoea* sp009829915	N/A^e^	sp009829915	Nvir^f^ (GCF_009829915.1)	None

^a^List of Prokaryotic names with Standing in Nomenclature (Parte et al., 2020).

^b^Genome Taxonomy Database (Hugenholtz et al., 2021) release 207.

^c^National Center for Biotechnology Information.

^T^Type material is a strain.

^e^Not applicable.

^Ts^Type material is a sequence.

^f^Genome was not included because of low genome quality, and, therefore, the species cluster was not used either.

**Figure 1:**
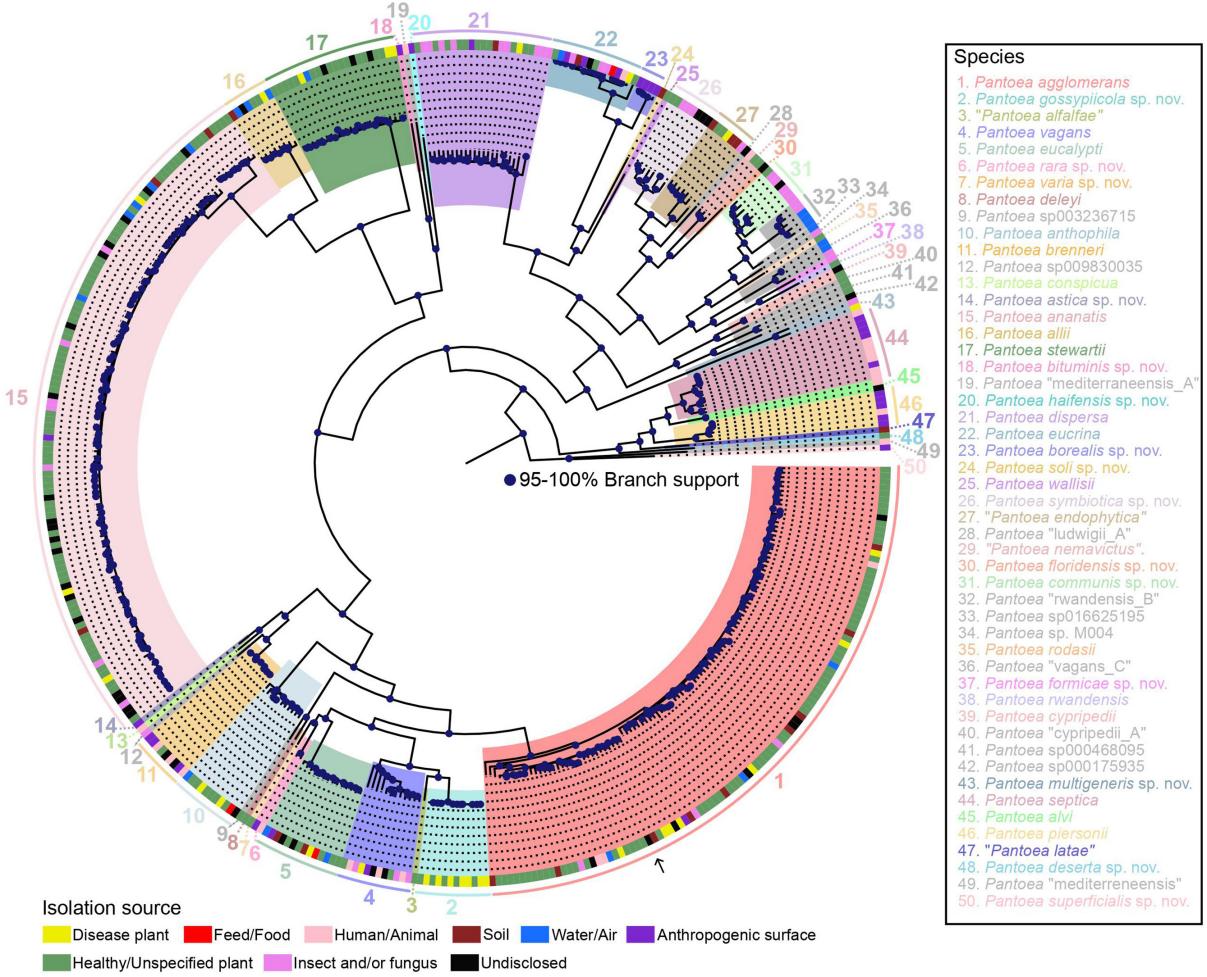
ML phylogeny of 437 representative genomes of the genus *Pantoea* based on the alignment of 2,509 core genes and rooted with *Mixta calida* DSM 22759^T^ (outgroup not shown). Species names (or GTDB species cluster identifiers for yet-unnamed species) as well as isolation sources are provided in the legend. Species newly named under the SeqCode are indicated with “sp. nov.” following the species epithet. The nomenclatural type for the genus, *P. agglomerans* DSM 3493^T^, is indicated with an arrow. Strain names were suppressed but are included in the same tree shown in Supplementary Figure 1, which also highlights type strains or type sequences, respectively, and includes within-species lineage information. Strain isolation sources from the NCBI BioSample database and other strain metadata are provided in Supplementary Table 1, which also includes the 122 genomes omitted from the tree because of high similarity to the genomes that were included.

The core genome and pangenome were then determined. The pangenome was found to be composed of 36,144 orthologous groups, and the core genome was found to consist of 2,509 orthologous groups (based on their presence in 95% of the analyzed genomes) with a total length of 2,737,734 nucleotides. From this data, two robust core genome trees were constructed using ML approaches, i.e., a ML tree constructed with IQ-TREE (Supplementary Figure 1) and an approximate ML tree constructed with FastTree (Supplementary Figure 2). A pangenome tree using all 36,144 orthologous genes was also constructed (Supplementary Figure 3). The ML core genome tree in which the major clades are highlighted is shown in Figure 1. There was overall high congruence among all three trees, and individual phylogenetic lineages are described below.

To identify population clusters in the genus *Pantoea*, the reverse ecology tool PopCOGent (Arevalo et al., 2019) was used to identify populations based on recent horizontal gene transfer (HGT) events. Genomes inferred to belong to the same populations were denoted with the same population identifiers (Supplementary Figure 1). In most cases, species contained two or more population clusters. Only a small number of species, mostly species for which only a few genomes have been sequenced, belonged to the same cluster (see details below).

Finally, strain source information available in NCBI's BioSample and BioProject databases and in the published literature were correlated with genome similarity, phylogeny, and ecology. Sources were classified into the following categories: healthy or unspecified plants, diseased plants, humans/animals, insects, soil, fungi, water/air, anthropogenic surfaces, feed/food, and undisclosed. Categories are listed for all individual genomes in Figure 1, Supplementary Figure 1, and Supplementary Table 1 and are summarized by species in Supplementary Table 2.

A detailed description of 49 *Pantoea* species follows in the order in which they are located in the tree in Figure 1 (clockwise, starting with *P. agglomerans*). For each species, taxonomic details (species name, etymology, type strain or genome, and/or representative genome in GTDB, and synonyms) are provided in Table 1, and LINs are provided in Supplementary Table 3. Yet-unnamed species clusters were named under the SeqCode (Hedlund et al., 2022; Whitman et al., 2022) when raw reads for at least one genome sequence, chosen as the type, were available in the NCBI SRA database, as required by the SeqCode. New species names are labeled with “sp. nov.” following species epithets.

### *Pantoea agglomerans* (76_A_6_B_2_C_0_D_0_E_3_F_)

#### Phylogeny, genomics, and reverse ecology

Out of 122 genomes identified as *P. agglomerans* (Gavini et al., 1989) in the GTDB Release 207, 104 representative genomes were used in our analysis (the other 17 genomes were omitted to limit redundancy since they had >99.95% ANI in the genomes that were included). The chosen genomes cluster with the genome of the *P. agglomerans*-type strain DSM 3493^T^ in a single clade with 100% bootstrap support. Genomes in this clade have a minimum pairwise ANI of 97%, indicating their conspecificity. All genomes in this clade are thus members of *P. agglomerans*, even though some are misidentified or mislabeled in NCBI as *P. stewartii, P. vagans*, or the non-validly published heterotypic synonyms “*P. pleuroti”* and “*Curtobacterium plantarum*” (Ma Y. et al., 2016) (see Supplementary Table 1 for NCBI organism names, GTDB names, and species names assigned based on our analysis). The ANI between the genome of DSM 3493^T^ and the most similar genome outside of the clade, that of strain DE0421 (*P. eucalypti*), is approximately 91%, confirming this taxon's genomic exclusivity from genomes in other clades. Genomes misidentified as *P. agglomerans* in NCBI are located in numerous other clades (see Supplementary Table 1).

The *P. agglomerans* clade is divided into three subclades (Figure 1). Clades 1 and 2 are fully supported in the ML analysis (Supplementary Figure 1). Clade 3 consists of a single genome, P5. The same subclades are present in the approximate ML core genome tree (Supplementary Figure 2) and in the pangenome tree (Supplementary Figure 3). Genomes in subclade 1 have a minimum pairwise ANI of 98.5%, and genomes in subclade 2 have a minimum pairwise ANI of 98.0%. The approximate ANI between the subclades is 98%. The subclades also represent distinct population clusters based on HGT, with subclade 2 including two genomes comprising the sole members of distinct and separate populations. When comparing the gene content of the subclades, we found 13 genes (encoding electron transport chain-related proteins, transporters, a carrier protein, and pilus/fimbriae-associated proteins) to be present in over 95% of subclade 2 genomes and absent from subclade 1 genome. Genome P5 contains 75 genes (encoding CRISPR-associated proteins, transporters, enzymes, and hypothetical proteins) not present in any of the genomes of subclades 1 and 2. However, no genes were present in over 95% of subclade 1 genomes and absent from subclade 2 genomes (Supplementary Table 4). Therefore, gene content differences do not strongly support distinct ecological differentiation of the identified subclades, which is also in agreement with the similar isolation sources reported for strains in the two clades.

The genome of strain *P. agglomerans* pv. *gypsophilae* 824-1 is located in *P. agglomerans* subclade 1, and the genome of *P. agglomerans* pv. *betae* 4188 is located in *P. agglomerans* subclade 2. These strains are representatives of a pathogen of baby's breath (*Gypsophila paniculata*) and of beet (*Beta vulgaris*), respectively. Since only one genome is available for each of the two pathogens, it is not possible to determine if the baby's breath pathogen is specific to subclade 1 and the beet pathogen is specific to subclade 2.

*P. agglomerans* genomes share a most recent common ancestor (MRCA) with genomes in a clade that includes *P. vagans, P. eucalypti*, and a number of genomes of other species listed below. *P. deleyi* splits off at a node immediately basal to this clade. Based on previous phylogenomic analysis (Palmer and Coutinho, 2022), the effectively published species “*Curtobacterium plantarum”* and “*P. pleuroti”* cluster with the *P. agglomerans-*type strains and are heterotypic synonyms of *P. agglomerans*.

#### Ecology, lifestyle, and biotechnological uses

Most genome-sequenced strains in the *P. agglomerans* species were isolated as epiphytes or endophytes from a broad range of plants (92/120; see Supplementary Table 1 for details). Out of these 92 strains, eight are listed in the NCBI BioSample database as having been isolated from plants with disease symptoms. However, it is not clear whether all these strains caused the observed symptoms. *P. agglomerans* is thus more likely to be a common plant-associated bacterium instead of a plant pathogen. The exceptions are bacteria in the pathovars *gypsophilae* and *betae* mentioned above, since the genomes of these bacteria contain plasmids encoding *hrp* type III secretion systems (T3SS) and effectors that were shown to be necessary for the formation of galls on their host plants (Nissan et al., 2019). Some strains of *P agglomeran*s have also been shown to cause disease on onion leaves and bulbs (Edens et al., 2006; Moloto et al., 2020). One isolate of *P. agglomerans* from a corn leaf (named *Erwinia herbicola* in the original publication, a heterotypic synonym of the species) was one of the very first bacteria found to have ice nucleation activity (INA) (Lindow et al., 1978). INA bacteria are hypothesized to travel through the water cycle and possibly contribute to precipitation (Failor et al., 2017). In fact, two of the genome-sequenced *P. agglomerans* strains were isolated from precipitation. Another eight strains were isolated from the soil. This is consistent with early reports on the presence of *P. agglomerans* in water (Gavini et al., 1989) and soil (Kageyama et al., 1992). On a regular basis, *P. agglomerans* has also been reported as a clinical isolate (Milanowski et al., 1995; Bujdáková et al., 2001; Koester et al., 2020; Lorenzi et al., 2022). However, in most cases, isolates were not genome sequenced, and their identity as *P. agglomerans* could not be confirmed. While the genome-sequenced *P. agglomerans*-type strain ICMP 12534^T^ was isolated from a human knee laceration and other genome-sequenced strains were isolated from the sputum of a cystic fibrosis (CF) patient and from a cervix (based on NCBI BioSample data; Supplementary Table 1), in none of the cases of human infection could the observed symptoms be unequivocally attributed to *P. agglomerans*; see also (Rezzonico et al., 2010, 2012a,b).

Biotechnologically relevant properties include the production of antibiotics (Smith et al., 2013; Smits et al., 2019; Robinson et al., 2020), biocontrol of plant diseases (Vanneste et al., 2002; Kim et al., 2012; Smits et al., 2015; Lorenzi et al., 2022), and many others. Another potential biotechnological application could be the use of *P. agglomerans* with INA in the production of frozen foods and in the control of insects by making larvae more susceptible to frost damage (Coutinho and Venter, 2009). In conclusion, the entire *P. agglomerans* species appears to have been adapted to life in association with plants, and its members survive well in various environments. Therefore, bacteria in this species may frequently migrate between plants, soil, and aquatic environments and occasionally colonize animals and humans. Without strong evidence for or against human pathogenicity of the species or within-species lineages and the absence of genomic signatures to predict pathogenicity (Lorenzi et al., 2022), implementation in biotechnological applications, in particular as a biocontrol organism, remains negatively affected.

### *Pantoea gossypiicola* sp. nov. (76_A_6_B_2_C_0_D_0_E_2_F_)

Based on the BioSample metadata available for genomes in this species (34 in total, 12 of them included in our analysis), all strains (including the GTDB representative and nomenclatural type B_8^Ts^) were recovered as epiphytes from diseased cotton leaves (*Gossypium hirsutum*). To determine if *P. gossypiicola* sp. nov. is a pathogen of cotton, Koch's postulates would need to be completed. The minimum pairwise ANI of genomes in this species is 99.25%. The high intraclade ANI shows that the diversity within the species cluster has been poorly sampled. Although three clonal lineages can be distinguished based on phylogeny (each with 100% branch support) and ANI, all strains of the species belong to the same population, suggesting that the clonal lineages have not adapted to separate ecological niches. *P. gossypiicola* sp. nov. shares an MRCA with “*Pantoea alfalfae”*.

### “*Pantoea alfalfae*” (76_A_6_B_2_C_0_D_0_E_0_F_)

This species had two known representatives in GTDB at the time genome sequences were downloaded for analysis: SRS89 (the only genome included in the tree), isolated from *Stevia rebaudiana* seeds, and a metagenome-assembled genome (MAG) of medium quality (80.3% completeness), UBA8265, recovered from a laboratory enrichment culture for *Salmonella*. SRS89 is misidentified in the NCBI as *P. vagans*. Recently, the species was effectively published as “*P. alfalfae*” with strain CQ10, isolated from alfalfa (*Medicago sativa*) seeds, as a proposed type strain (Yao et al., 2023). The conspecificity of CQ10 and SRS89 is supported by phylogenomics in the recent release of GTDB. As stated above, “*Pantoea alfalfae”* shares an MRCA with *P. gossypiicola* sp. nov.

### *Pantoea vagans* (76_A_6_B_2_C_0_D_0_E_1_F_)

#### Phylogeny, genomics, and reverse ecology

*P. vagans* (Brady et al., 2009) includes the genomes of 13 strains, including the type strain LMG 24199^T^. One additional genome of the species was omitted from the phylogenetic analysis. In NCBI, these strains are either identified as *P. vagans* or only at the genus level. Strains misidentified in the NCBI as *P. vagans* are located in the phylogenetic tree as members of other species, for example, *P. agglomerans* and *P. eucalypti*. *P. vagans* genomes have a minimal pairwise ANI of 96%, revealing a genomically diverse group of genome-sequenced strains. This diversity is also reflected in the reverse ecology analysis, which identified six population clusters. One cluster (cluster 24, corresponding to subclade 1 in Supplementary Figure 1) has eight members, and the other four clusters only have one member each. Gene content comparison revealed that the genomes in population cluster 24 share 11 genes (encoding a type II toxin–antitoxin system CcdA family antitoxin and a hemolysin activator protein) that are absent from the other four genomes (Supplementary Table 4). Furthermore, the clade corresponding to population cluster 24 is also conserved in the other trees, further suggesting that population cluster 24 represents an ecologically distinct population (Supplementary Figures 2–4). The strains in the *P. vagans* clade share an MRCA with *P. gossypiicola* sp. nov. and “*P. alfalfae*” described above.

#### Ecology, lifestyle, and biotechnological uses

The *P. vagans*-type strain and other non–genome-sequenced strains of the species were isolated from eucalyptus presenting bacterial blight and dieback symptoms and from maize with brown stalk rot (Brady et al., 2009), but there is no evidence of being the causal agent of the observed disease symptoms. *P. vagans* has also been isolated from other plants, insects, and occasionally from humans in a medical context. However, as for the plant isolates, there is no indication that *P. vagans* caused these infections.

One example of the beneficial and biotechnological properties of *P. vagans* is the biocontrol organism *P. vagans* C9-1 (Ishimaru et al., 1988; Smits et al., 2011), which produces three antibiotics: the peptide-derived antibiotic Pantocin A (Smits et al., 2019), the antibiotic herbicolin I (Kamber et al., 2012a), and a third antibiotic, recently identified as PNP-3 (Williams and Stavrinides, 2020), which is an antibiotic not produced by any other strain of *P. vagans* currently sequenced (Sulja et al., 2022).

### *Pantoea eucalypti* (76_A_6_B_2_C_0_D_0_E_6_F_)

#### Phylogeny, genomics, and reverse ecology

The 15 genomes of *P. eucalypti* (14 of which were used in the phylogenetic analysis, including the genome of the type strain LMG 24197^T^) have a minimal pairwise ANI of over 99% and belong to the same population cluster, revealing that the *P. eucalypti* strains sequenced to date are members of a single clonal lineage. Some of these members are mislabeled in NCBI as they were misidentified as other *Pantoea* species, but their very high ANI to the *P. eucalypti*-type strain genome and their phylogenetic position in the same clade clearly identify them as *P. eucalypti*. The *P. eucalypti* clade shares an MRCA with *P. rara* sp. nov. and *P. varia* sp. nov. Recently, it was reported (Palmer and Coutinho, 2022) that the “*Pantoea hericii”*-type strain represents a heterotypic synonym of *P. eucalypti*.

#### Ecology, lifestyle, and biotechnological uses

The *P. eucalypti*-type strain was isolated from a eucalyptus plant with symptoms of blight and dieback in Uruguay (Brady et al., 2009). *P. eucalypti* was also isolated from other plant species, but no information is available about whether these isolates caused symptoms in any of these plants, and a T3SS gene cluster was not detected in any of the eight *P. eucalypti* genomes previously analyzed by Moretti et al. (2021). Other genome-sequenced *P. eucalypti* strains were isolated from precipitation, soil, and commercial poultry feed. Beneficial and biotechnological properties of *P. eucalypti* were described for *P. eucalypti* FBS 135, which promotes Masson pine (*Pinus massoniana*) seedling growth and increases seedling survival rates (Song et al., 2020).

### *Pantoea rara* sp. nov. (76_A_6_B_2_C_0_D_0_E_5_F_)

This species consists of the genomes of strain WMus005 isolated from the oral cavity of a wild house mouse (*Mus musculus*) and the metagenome-assembled genome (MAG) UBA5707^Ts^, chosen as the nomenclatural type, recovered from a metagenomic sample taken from the New York City subway system. The pairwise ANI of the two genomes is over 99%, and they thus clearly belong to the same species. *P. rara* sp. nov. shares an MRCA with *P. varia* sp. nov.

### *Pantoea varia* sp. nov. (76_A_6_B_2_C_0_D_0_E_4_F_)

This species consists of the genomes of two strains, of which OV426^Ts^ was chosen as the nomenclatural type, isolated during the characterization of *Populus* root and rhizosphere microbial communities and from an indoor dust sample (the genome of this strain, AF015A5, is not included in the GTDB and not in our analysis but is deposited in the RefSeq database with accession number GCF_004798195.1 and misidentified as *P. allii*). The two genomes have a pairwise ANI of approximately 97%. As stated above, *P. varia* sp. nov. shares an MRCA with *P. rara* sp. nov.

### *Pantoea deleyi* (76_A_6_B_2_C_0_D_1_E_0_F_)

The genome of the *P. deleyi*-type strain, LMG 24200^T^, the only genome of this species included in our analysis, shares an MRCA with *P*. sp003236715. The *P. deleyi*-type strain was isolated from *Eucalyptus* (Brady et al., 2009). The symptoms of infection on leaves and shoots include bacterial blight and dieback. The genome of *P. deleyi* contains two gene clusters encoding T3SS: an uncharacterized Hrp-2b type and the SPI-1 type, highly similar to the SPI T3SSs of the plant pathogenic strains *P. stewartii* subsp. *stewartii* DC283 or *E. amylovora* CFBP 1430 (Smits et al., 2010; Correa et al., 2012; Kamber et al., 2012b; Moretti et al., 2021), suggesting that the symptoms observed on the host plant may have been caused by *P. deleyi*.

### *Pantoea* sp003236715 (76_A_6_B_2_C_0_D_1_E_1_F_)

In GTDB release 207, this species cluster encompasses a single genome-sequenced strain, ARC607, isolated from rice (*Oryza sativa*) seeds. As mentioned above, this species shares an MRCA with *P. deleyi*.

### *Pantoea anthophila* (76_A_6_B_2_C_0_D_3_E_0_F_)

*P. anthophila* consists of the genome of the type strain LMG 2558^T^ and 13 additional genomes (three of which were omitted from the phylogenetic analysis). The *P. anthophila* clade splits at a node that is basal to all clades described to date. It includes strains that are misidentified or mislabeled as *P. agglomerans* and *P. vagans* in the NCBI. However, the minimal pairwise ANI of strains within the clade is 98%, clearly showing that all members belong to *P. anthophila*. There are seven separate population clusters in this species. The four genomes that belong to the same population share seven genes (including, among others, genes coding for bacteriocin and hypothetical proteins) that are absent from the seven genomes in the other six clusters (Supplementary Table 4), supporting the idea that this population may occupy a separate ecological niche. However, this is not evident based on isolation source information since the majority of strains in the species, including the type strain, were isolated from plants. There is currently no indication of pathogenicity, and only three out of seven *P. anthophila* genomes analyzed by Moretti et al. (2021) had a gene cluster coding for a T3SS. Furthermore, one *P. antophila* strain was isolated from hypersaline lake water and one from commercial poultry feed, suggesting that, similar to *P. agglomerans* and *P. vagans*, this species may not only be adapted to life in association with plants but also survive well as a free-living organism in the environment.

### *Pantoea brenneri* (76_A_6_B_2_C_0_D_2_E_2_F_)

#### Phylogeny, genomics, and reverse ecology

Nine genomes of *P. brenneri* were used in the phylogenetic analysis, including the genome of the type strain LMG 5343^T^, and another 15 genomes were not included. The minimal pairwise ANI in the *P. brenneri* clade is 99%, suggesting that this species has limited diversity or that sampling has been limited. This is supported by reverse ecology analysis, which assigned all genomes in the clade to the same population cluster. The genome most closely related to *P. brenneri* is that of strain Eser (*P*. sp009830035), with which the *P. brenneri* clade shares an MRCA.

#### Ecology, lifestyle, and biotechnological uses

The type strain, *P. brenneri*, was isolated from a human urethra. Another two strains were isolated from human sputum and an unidentified source in Canada (Brady et al., 2010). Sixteen (two of them included in the phylogenetic analysis) almost identical genome-sequenced *P. brenneri* strains were isolated from environmental surfaces on the International Space Station. Other genome-sequenced strains were isolated from human samples and anthropogenic environments, including a sample of brown algae (*Ectocarpus subulatus*) and an endophyte sample from gray willow (*Salix atrocinerea*). Overall, *P. brenneri* appears to have a much closer association with humans and is more commonly found in anthropogenic environments than *P. agglomerans, P. vagans*, and *P. anthophila* (among others). Whether it is a human commensal or an opportunistic human pathogen cannot be concluded at this point.

### *Pantoea* sp009830035 (76_A_6_B_2_C_0_D_2_E_3_F_)

This species cluster consists of the genome of a single strain, Eser, isolated from a brown stink bug (*Euschistus servus*). It shares an MRCA with the *P. brenneri* clade (see above).

### *Pantoea conspicua* (76_A_6_B_2_C_0_D_2_E_1_F_)

The only genome in the *P. conspicua* clade is of the species-type strain LMG 24534^T^. It shares an MRCA with *Pantoea astica* sp. nov. The type strain was isolated from human blood (Brady et al., 2010).

### *Pantoea astica* sp. nov. (76_A_6_B_2_C_0_D_2_E_0_F_)

This species consists of a single MAG, UBA6564^Ts^ in GTDB release 207, which was chosen as a nomenclatural type. It was assembled from a DNA sample of a metal surface in New York City. *P. conspicua* is the most closely related species (see above).

### *Pantoea ananatis* (76_A_6_B_13_C_0_D_1_E_0_F_)

#### Phylogeny, genomics, and reverse ecology

The 114 genomes in the *P. ananatis* species that were used in the phylogenetic analysis, including the genome of the type strain LMG 2665^T^, have a minimal pairwise ANI of 96%. Another 35 genomes were omitted because they were highly similar to the 114 genomes that were included. The *P. ananatis* clade is divided into two main subclades, each fully supported in core genome phylogenies (labeled 1 and 2 in Supplementary Figure 1) and also maintained in the pangenome tree (Supplementary Figure 3). The minimal pairwise ANI is 98.5% for genomes within subclade 1 and 99% for genomes within subclade 2. The ANI between the two subclades is ~97%. The two subclades represent two separate population clusters, with subclade 1 strain FDAARGOS680 being the only member of a third population. This suggests that strains in the two subclades may occupy separate ecological niches. This is also supported by gene content differences. Two genes (one related to ion transport and a putative beta-carotene dioxygenase) are present in over 95% of subclade 1 genomes, but neither in a single genome of subclade 2 nor FDAARGOS680. Additionally, nine genes (associated with cytochrome C, membrane proteins, and hypothetical proteins) are present in over 95% of subclade 2 genomes but completely absent from subclade 1 genomes and from FDAARGOS680. The genome of strain FDAARGOS680 contains 160 genes not present in any of the genomes of subclades 1 and 2 (Supplementary Table 4). *P. ananatis* shares a MRCA with *P. allii*. We note that some genomes in the *P. ananatis* clade are misidentified or mislabeled in NCBI as *P. vagans*, while other genomes located in the phylogenetic tree in clades corresponding to other species are misidentified or mislabeled as *P. ananatis* in NCBI.

#### Ecology, lifestyle, and biotechnological uses

*P. ananatis* LMG 2665^T^ was isolated from the diseased *Ananas comosus* (pineapple). Bacteria in the *P. ananatis* species have been isolated over the years as pathogens from a growing number of crops and forest plants (Coutinho and Venter, 2009), for example, as a causative agent of the bacterial leaf blight of rice (Yu et al., 2021). *P. ananatis* has received extensive attention as a pathogen of onions, where its capacity to cause disease is dependent on the HiVir biosynthetic genes for the phosphonate phytotoxin Pantaphos. *P. ananatis* onion virulence is also supported by *alt* (allicin tolerance) genes conferring increased resistance to *Allium* thiosulfinate defensive chemistry (Asselin et al., 2018; Stice et al., 2020; Agarwal et al., 2021a; Polidore et al., 2021; Shin et al., 2023). *P. ananatis* has also been isolated as an endophyte and an epiphyte from many healthy plants, a symptomatic button mushroom, insects, soil, precipitation, and an air sample. It was also reported to cause bacteremia in humans, but identification was only based on partial 16S rRNA sequencing (De Baere et al., 2004); thus, the taxonomic affiliation of these strains remains uncertain. Not a single genome-sequenced isolate originates from human tissues or organs. Therefore, isolation sources and pathogenicity assays of *P. ananatis* clearly indicate pathogenic potential in plants, but pathogenicity in humans and animals remains uncertain.

There are several examples of biotechnological applications of *P. ananatis*. For example, a genetically engineered *P. ananatis* strain was used to synthesize the amino acids cysteine (Takumi et al., 2017) and L-glutamic acid (Hara et al., 2012) and isoprenoids (Nitta et al., 2021). Ma J. et al. (2016) demonstrated that another *P. ananatis* strain metabolizes rice straw by degrading lignocellulose, and Schulz et al. (2017) showed that *P. ananatis*, in the presence of the yeast *Papiliotrema baii*, detoxifies hydroxylated benzoxaxolinones. Deivanai et al. (2014) instead described that rice seedlings have higher fitness in the presence of *P. ananatis* 13 SE 1, and Mechan Llontop et al. (2020) found a strain isolated from rain to have biocontrol activity against the fire blight pathogen *E. amylovora*. Furthermore, the *crtI* carotene desaturase gene of *P. ananatis* (referred to in the publication under its heterotypic synonym *Erwinia uredovora*) was incorporated into the vitamin A biosynthetic pathway of Golden Rice (Paine et al., 2005).

### *Pantoea allii* (76_A_6_B_13_C_0_D_0_E_0_F_)

#### Phylogeny, genomics, and reverse ecology

The eight genomes in the *P. allii* clade, including that of the type strain LMG 24248^T^, have a minimal pairwise ANI of at least 98.5%, revealing genomic homogeneity significantly above the 95% ANI species threshold. However, the clade has a substructure well-supported by the ML phylogeny, and genomes were assigned to three distinct population clusters consisting of four genomes, three genomes, and one genome, respectively, possibly suggesting the occupation of separate ecological niches. However, this was not supported by the other trees (Supplementary Figures 2, 3), which did not maintain the same clade substructure, and was only partially supported by differences in gene content: no genes were found to be uniquely present in the largest population, 14 genes were found to be uniquely present in the second population, and 153 genes were found to be present only in the genome of strain BAV3077 (Supplementary Table 4). *P. allii* shares an MRCA with *P. ananatis* and *P. stewartii*.

#### Ecology, lifestyle, and biotechnological uses

The *Pantoea allii-*type strain was isolated from onion seed (*Allium cepa*) (Brady et al., 2011). The capacity of the type strain to cause necrosis on onion leaves was shown to be dependent on the halophos phosphonate biosynthetic gene cluster (Zhao et al., 2023). Other *P. allii* strains were isolated from onions with center rot (Brady et al., 2011). Genome-sequenced *P. allii* strains were also isolated from the seed and rhizosphere soils of other plants and from rainfall. While *P. allii* is a confirmed onion pathogen, it is not yet clear if it causes disease on any of the other plants from which it was isolated.

### *Pantoea stewartii* (76_A_6_B_13_C_1_D_0_E_0_F_)

#### Phylogeny, comparative genomics, and reverse ecology

The *P. stewartii* clade consists of 22 genomes, including the genome of the type strain CCUG 26359^T^, while another 11 genomes were omitted from the phylogenetic analysis. The *P. stewartii* clade splits off at a node immediately basal to *P. allii* and *P. ananatis*. The maximal pairwise ANI among genomes within the clade is 98.5%, revealing limited genomic diversity. The clade has an extensive substructure with a dozen small clades that are highly supported by all phylogenies. However, these clades do not correlate with the six population clusters identified by reverse ecology analysis. In particular, population cluster 4 includes the genomes of many separate clades, which could be explained by frequent recombination events.

The assignment of genomes to subspecies *stewartii*, the pathogen that causes Stewart's Wilt of corn (*Zea mays*), and subspecies *indologenes*, the pathogen described as the causative agent of leaf spot on millet (Mergaert et al., 1993), is incomplete in NCBI. Furthermore, based on isolation sources reported in NCBI's BioSample database, neither the genomes of strains recovered from corn nor the genomes of strains recovered from millet can be assigned to separate clades. Moreover, these genomes are interspersed with the genomes of strains recovered from other plants and the environment. Therefore, based on isolation sources alone, neither subspecies appears to be monophyletic. Further studies will be necessary to clarify how subspecies are related to each other, including the use of discriminatory primers (Shin et al., 2022).

#### Ecology, lifestyle, and biotechnological uses

Stewart's Wilt disease was first described by Stewart (1897), and the causative agent was later described as *P. stewartii* subsp. *stewartii* (Mergaert et al., 1993), which is transmitted by the corn flea beetle (*Chaetocnema pulicaria*) (Rand and Cash, 1937). *P. stewartii* subsp. *indologenes* was originally described as a pathogen of leaf spot on pearl millet (*Pennisetum glaucum*) and foxtail millet (*Setaria italica*) (Mergaert et al., 1993) but, since then, it has been found to cause disease on several additional plant species, including onion (*Allium cepa*) (Agarwal et al., 2021b; Koirala et al., 2021). *P. stewartii* subsp. *stewartii* is a T3SS-dependent pathogen of corn. After identifying subspecies *indologenes* as pathogens of center rot of onion (*Allium cepa*) and other *Allium* species (Stumpf et al., 2018), two pathovars were described, cepacicola and setariae, based on their capacity to cause disease on both millet and *Allium* species or only on millet, respectively (Koirala et al., 2021). While *P. stewartii* subsp. *indologenes* pv. cepacicola pathogenicity on millet is T3SS-dependent, pathogenicity on onion is dependent on either the HiVir or halophos phosphonate biosynthetic gene clusters (Zhao et al., 2023). Based on NCBI BioSample information, *P. stewartii* was also isolated from several additional plant species, a waterfall, and an unspecified environmental source. In most cases, the NCBI BioSample data do not indicate if the plants were diseased, but several publications confirm that *P. stewartii* is in fact a pathogen of these plants (Azizi et al., 2019; Abidin et al., 2021). We conclude that *P. stewartii* as a species is potentially a pathogen of a wide range of plants but that more host range testing, similar to the study published by Koirala et al. (2021), needs to be done to understand the degree to which different lineages within the species adapt to different plant species.

### *Pantoea bituminis* sp. nov. (76_A_6_B_2_C_3_D_0_E_0_F_)

In GTDB release 207, this species consists of the genome of a single strain, EnvD^Ts^, the nomenclatural type, which was isolated from asphalt. *P*. “mediterraneensis_A” is the most closely related species cluster (see below).

### *Pantoea* “mediterraneensis_A” (76_A_6_B_2_C_2_D_0_E_0_F_)

This species cluster was effectively published as “*Mixta mediterraneensis*” (Boxberger et al., 2021). However, both our phylogenetic analysis and GTDB place this taxon in the genus *Pantoea*. The species cluster currently consists of the genome of a single strain, Marseille-Q2057, which was isolated from the skin swabs of a healthy human (Ndiaye et al., 2019; Boxberger et al., 2021). As stated above, *P*. “mediterraneensis_A” shares an MRCA with *P. bituminis* sp. nov.

### *Pantoea haifensis* sp. nov. (76_A_6_B_2_C_4_D_0_E_0_F_)

This species currently consists of the genome of a single strain, EnvH^Ts^, the nomenclatural type, which was isolated from asphalt in Haifa, Israel. *P. haifensis* sp. nov., *P*. “mediterraneensis_A”, and *P. bituminis* sp. nov. share an MRCA that splits off at a node basal to all clades described to date.

### *Pantoea dispersa* (76_A_6_B_5_C_3_D_0_E_0_F_)

#### Phylogeny, genomics, and reverse ecology

The clade corresponding to *P. dispersa* consists of 23 genomes, including the genome of the type strain DSM 30073^T^ (with an additional 12 genomes that were not included in the phylogenetic analysis). The clade shares an MRCA with a clade corresponding to *P. eucrina, P. borealis* sp. nov., *P. wallisii*, and *P. soli* sp. nov. The minimal pairwise ANI among *P. dispersa* genomes is 96%. Within *P. dispersa*, many subclades can be seen; however, many of them are unsupported and inconsistent across trees. Only one subclade, consisting of two genomes, is clearly distinct from the other subclades based on ANI since these two genomes have an ANI below 97% for all other *P. dispersa* genomes.

#### Ecology, lifestyle, and biotechnological uses

Genome-sequenced *P. dispersa* strains have been isolated from sources as diverse as soil, plants, insects (including fungal gardens of fungus-farming ants), a fermentation starter, and a nursing call button in a hospital intensive care unit. There are also reports of *P. dispersa* as a potential pathogen of humans and plants (Yang et al., 2023) but, because identification was limited to partial 16S rRNA sequencing and/or Koch's postulates were not completed, any conclusions about the pathogenicity of *P. dispersa* to plants, animals, and humans would be premature. Biotechnological properties of *P. dispersa*, again identified based on partial 16S rRNA sequencing, include the growth promotion of sugarcane (*Saccharum*) (Singh et al., 2020).

### *Pantoea eucrina* (76_A_6_B_5_C_5_D_0_E_1_F_)

#### Phylogeny, genomics, and reverse ecology

The *P. eucrina* clade consists of 15 genomes, including the genome of the type strain LMG 5346^T^ (plus one genome that was omitted). The clade shares an MRCA with *P. borealis* sp. nov. The maximal pairwise ANI among *P. eucrina* genomes is ~96%, showing that the diversity within this species has been sampled more broadly than many other *Pantoea* species. Within the species, it is possible to distinguish four clades, each fully supported in the core genome phylogenies (Supplementary Figures 1, 2) and also present in the pangenome tree (Supplementary Figure 3). Three of the clades have multiple members with pairwise ANI within each clade above 98%. Clade 4 is presented by a single strain, M_9. Based on reverse ecology analysis, the genomes of the first three clades belong to the same population cluster, and only the single genome of clade 4 represents a separate population. No genes were found to distinguish the clusters. This suggests that, although based on ANI and phylogeny *P. eucrina* appears diverse, its analyzed members share a similar ecological niche.

#### Ecology, lifestyle, and biotechnological uses

The *P. eucrina*-type strain was isolated from a human trachea. Brady et al. (2010) also reported isolations from a human cyst, human urine, and human spinal fluid. Genome-sequenced strains were isolated from a wide variety of sources, including plants, a fungal garden of a fungus-farming ant, a mosquito, precipitation, and even a ready-to-eat vegetable soup in a nursing home. Therefore, *P. eucrina* strains appear to survive well in many environments without having adapted to any specific environment. While the species description includes several human-associated sources, only one of the genome-sequenced strains is human-associated. Two plant sources are listed as symptomatic, but *P. eucrina* is not described as the causative agent. Therefore, it is premature to draw any conclusions about the risk that bacteria in this species are opportunistic human or plant pathogens.

Biotechnological properties involve a strain identified as *P. eucrina* based on 16S rRNA sequencing that was isolated from a marine sponge (*Chondrosia reniformis*), which was found to produce N-lipoamino acids, some with potential antimicrobial activity (Vitale et al., 2020); however, more robust identification of the strain is required to confirm its taxonomic affiliation.

### *Pantoea borealis* sp. nov. (76_A_6_B_5_C_5_D_0_E_0_F_)

Four genomes of this species were included in our analysis. The genome of the MAG UBA6694^Ts^, the nomenclatural type, and three other MAGs were sourced from environmental samples of metallic or plastic surfaces of the New York City subway. The fourth genome is of the strain Russ, isolated from an indoor trash can. Pairwise ANI among the four genomes is over 99.25%, revealing that they belong to a single clonal lineage. The *P. borealis* clade shares an MRCA with the *P. eucrina* clade. One publication about the strain Russ discusses its potential for being an opportunistic human pathogen (Moghadam et al., 2016). Other genomes have not been described in the literature. Therefore, at this point, the species appears prevalent in anthropogenic environments, but there is no evidence for it being a pathogen.

### *Pantoea soli* sp. nov. (76_A_6_B_5_C_4_D_0_E_0_F_)

The genome of a single strain, DSM 32899^Ts^, which is the nomenclatural type, belongs to the species *P. soli*. The type strain was isolated from the soil. It splits off at a node that is basal to the *P. eucrina*/*P. borealis* clade.

### *Pantoea wallisii* (76_A_6_B_5_C_6_D_0_E_0_F_)

The genome of the only sequenced strain of *P. wallisii*, type strain LMG 26277^T^, is located on a branch that splits off basally to form *P. soli* sp. nov. The *P. wallisii*-type strain was isolated from diseased *Eucalyptus* seedlings, but there was no evidence that it caused the observed disease symptoms (Brady et al., 2012).

### *Pantoea symbiotica* sp. nov. (76_A_6_B_5_C_1_D_0_E_2_F_)

This species includes nine genomes, one of which is the nomenclatural type YR512^Ts^. Another two genomes were not included in the phylogenetic analysis. The clade corresponding to this species is a sister clade of “*P. endophytica”*. Pairwise ANI between the eight genomes is over 97%. The strains were isolated from tree roots (Brown et al., 2012), fungi (Wong et al., 2020), and endophytes of grapevine. Based on these isolation sources, this species appears to be adapted to the rhizosphere.

### “*Pantoea endophytica*” (76_A_6_B_5_C_1_D_0_E_1_F_)

This effectively published species, “*P. endophytica*” (Gao et al., 2019), consists of six genomes, including that of the type strain 596^T^. The genomes have a reciprocal ANI of over 98%, and one more divergent genome, B9002, is basal to the other genomes and has an ANI of ~96% to the former. The same subclade structure is found in all phylogenies (Supplementary Figures 2, 3). The six highly similar genomes belong to three distinct population clusters, and the more divergent genome is the only member of a fourth population. Comparing gene content revealed that these population clusters contain 4, 9, 255, and 545 unique genes, respectively, further suggesting that they may have adapted to different ecological niches (Supplementary Table 4). “*P. endophytica*” shares an MRCA with *P. symbiotica* sp. nov. “*P. endophytica*” strains have been isolated from plant roots and soil. Based on the small sample of genome-sequenced strains, this species may thus be mainly plant root- and soil-associated.

### *Pantoea* “ludwigii_A” (76_A_6_B_5_C_1_D_0_E_0_F_)

This species cluster consists of the genome of a single strain, EnVs2, which was isolated from a grapevine (*Vitis vinifera*) stem (Lòpez-Fernàndez et al., 2016). In the core genome tree, the genome of EnVs2 is located on a branch that shares an MRCA with the MRCA of the clade that includes “*P. endophytica*” and *P. symbiotica* sp. nov.

### “*Pantoea nemavictus*” (76_A_6_B_5_C_1_D_2_E_0_F_)

This effectively published species (Dirksen et al., 2020) currently consists of the genomes of three strains, including the genome of the type strain BIGb0393^T^, with a pairwise ANI of over 98%. The corresponding clade splits off at a node that is basal to the *P. symbiotica* sp. nov. and “*P. endophytica*” clades. Isolation sources are a roundworm (*Caenorhabditis elegans*) from a rotting plant stem, the internal stem tissue of poison ivy (*Toxicodendron radicans*), and poplar (*Populus deltoides*) roots. This species may thus be a plant endophyte.

### *Pantoea floridensis* sp. nov. (76_A_6_B_5_C_1_D_1_E_0_F_)

The clade corresponding to this species consists of the genome of a single strain, JKS000234^Ts^, chosen as the nomenclatural type. It was isolated from a fungus-farming ant garden in Florida, USA. One additional, very similar genome from the same source was omitted from the tree. This species shares an MRCA with “*P. nemavictus”*.

### *Pantoea communis* sp. nov. (76_A_6_B_5_C_0_D_1_E_0_F_)

This species consists of seven genome-sequenced strains, including the nomenclatural type Al-1710^Ts^. The minimal pairwise ANI among the seven genomes is 97%. The species shares an MRCA with the genome of *P*. “rwandensis_B”. Isolation sources include insects, an ant fungus garden, plants, and other environmental sources. Therefore, this species shares plants and insects as isolation sources with many other *Pantoea* species, and no adaptation to any specific environment is evident.

Note: There is an eighth genome in the same LINgroup as *P. communis* sp. nov., M004, which is the GTDB representative of the corresponding species cluster “*Pantoea* cancerogena_A” and is identified as *Enterobacter cancerogenus* in NCBI, isolated from a waterfall. This genome is located on a branch that splits off at a basal node to the *P*. “rwandensis_B” clade in the core genome tree (in both the IQ-Tree and FastTree), although it has an ANI of over 95% with the seven *P. communis* sp. nov. genomes and clusters with the other seven *P. communis* sp. nov. genomes in the same clade with 100% branch support in the pangenome tree (Supplementary Figure 3). This is a rare case where ANI and core genome phylogeny do not correlate with each other. Therefore, although this genome shares a high ANI with members of *P. communis* sp. nov., the assignment of this genome to any species remains uncertain as phylogeny and genome metrics are not in agreement.

### *Pantoea* “rwandensis_B” (76_A_6_B_5_C_0_D_1_E_2_F_)

Five genome-sequenced strains belong to this species cluster, including the GTDB representative ND04. Pairwise ANI among the five genomes is 98% or higher. As described above, the clade corresponding to this species shares an MRCA with seven of the *Pantoea communis* sp. nov. genomes. Isolation sources of *P*. “rwandensis_B” include the coffee berry borer (*Hypothenemus hampei*), a river, a natural lake, and a waterfall. Therefore, this species may be more adapted to aquatic environments compared to other *Pantoea* species.

### *Pantoea* sp016625195 (76_A_6_B_5_C_0_D_1_E_1_F_)

Only the genome of a single strain, GTDB representative S63, belongs to this species cluster. Strain S63 was isolated from the coffee borer beetle (*Hypothenemus hampei*). The genome is located on a branch that splits off at a node that is basal to the *P*. “rwandensis_B”/*P. communis* sp. nov. clade.

### *Pantoea rodasii* (76_A_6_B_5_C_0_D_3_E_0_F_)

The *P. rodasii* clade is represented by the single genome sequence of the type strain LMG 26273^T^ and is located on the branch splitting off at the most basal node of the *P*. “rwandensis_B”/*P. comunis* sp. nov.*/P*. sp016625195 clade. The type strain was isolated from *Eucalyptus* leaves with bacterial blight and dieback symptoms. However, based on the data provided in the species description (Brady et al., 2012), it is not clear if it was the causative agent of the observed symptoms.

### *Pantoea* “vagans_C” (76_A_6_B_5_C_0_D_0_E_0_F_)

This species cluster consists of three genome-sequenced strains: ND02 (the GTDB representative) and ND03, both isolated from a waterfall located in a tropical rainforest, and Ps-ISGKm56, obtained from the midgut of a stink bug. These genomes share an ANI of over 97%. Note that genome ND03 is designated in NCBI as *P. rodasii*. In the core genome tree, the clade corresponding to this group is a sister to the *P. rwandensis*/*P. formicae* sp. nov. clade (see below).

### *Pantoea formicae* sp. nov. (76_A_6_B_5_C_0_D_2_E_0_F_)

This species is represented by the genome of a single strain, Acro-805^Ts^, the nomenclatural type, isolated from the fungal garden of an ant. It shares an MRCA with the type strain of *P. rwandensis* (see below).

### *Pantoea rwandensis* (76_A_6_B_5_C_0_D_2_E_1_F_)

The genome of the type strain, LMG 26275^T^, is the only available genome sequence for this species. It shares an MRCA with the *P. formicae* strain Acro-805. Bacteria in the *P. rwandensis* species, including the type strain, were isolated from *Eucalyptus* seedlings with bacterial blight or dieback symptoms (Brady et al., 2012), but based on the species description, it is not clear if they represent the causative agent.

### *Pantoea cypripedii* (76_A_6_B_5_C_7_D_0_E_1_F_)

The *P. cypripedii* clade consists of the type strain LMG 2657^T^ and one other strain of unknown origin, which has an ANI of over 99.9% compared to the type strain. The two genomes, which are both in the tree, also belong to the same population cluster. *P. cypripedii* shares an MRCA with the species cluster *P*. “cypripedii_A”. The *P. cypripedii*-type strain was isolated from a Lady Slipper Orchid (*Cypripedium*) (Brady et al., 2010), but there is scarce knowledge about the ecology and life cycle of this species.

### *Pantoea* “cypripedii_A” (76_A_6_B_5_C_7_D_0_E_0_F_)

This species consists of a single genome-sequenced strain, NE1 (the GTDB representative), isolated as an endophyte from an Egyptian Riverhemp (*Sesbania* spp.) root nodule and found to be plant growth promoting based on the provided metadata.

### *Pantoea* sp000468095 (76_A_6_B_5_C_7_D_1_E_1_F_)

This species cluster consists of the genomes of two strains, AS-PWVM4 (the GTDB representative) and MSR2. ANI between the two genomes is above 98.5%. The corresponding clade shares an MRCA with the GTDB species cluster *P*. sp000175935. Strain AS-PWVM4 was isolated from a plant rhizosphere sample and found to have biofertilizer and biocontrol activities (Khatri et al., 2013), while MSR2 was obtained from the roots of a legume plant. This species cluster may thus be a root endophyte based on the two available genomes.

### *Pantoea* sp000175935 (76_A_6_B_5_C_7_D_1_E_0_F_)

This species consists of the genome of a single strain, At-9b, the GTDB representative. Based on the genome's BioSample data, it was isolated from a corn (*Zea mays*) plant with Stewart's Wilt symptoms.

### *Pantoea multigeneris* sp. nov. (76_A_6_B_34_C_0_D_0_E_0_F_)

This species consists of the genomes of two strains, A4 and the nomenclatural type Acro-835^Ts^, which have a pairwise ANI of ~96%. Strain A4 was isolated from a decaying flower bud, while strain Acro-835 was isolated from an ant fungus garden. This species is located on the branch splitting off at the most basal node of the large clade that includes the species from *P. dispersa* to *P*. sp000175935 described above.

### *Pantoea septica* (76_A_6_B_2_C_1_D_1_E_1_F_)

#### Phylogeny, genomics, and reverse ecology

The 13 genomes (six of them MAGs) in this species (12 of them were included in the phylogenetic analysis) have pairwise genome similarity as low as 95% ANI, revealing a genomically diverse species. The corresponding clade consists of four subclades, each consisting of two to five genomes. Each clade is supported in all trees and corresponds to a separate population cluster, suggesting that strains of *P. septica* may possibly occupy at least four different ecological niches. Gene content analysis found 15, 50, 35, and 47 genes, respectively, to be unique to each subclade, further supporting that these are ecologically relevant populations. Genes unique to the four clades include genes coding for transporters, regulators, various enzymes, type VI secretion systems, flagella, chemotaxis, and hypothetical proteins (Supplementary Table 4).

#### Ecology, lifestyle, and biotechnological uses

The type strain of *P. septica*, LMG 5345^T^, was isolated from a human stool sample. Other strains were isolated from the human blood, skin, and gut, including one isolated from a preterm neonatal blood sepsis patient and one isolated from a human host sourced by an intensive care unit. The six MAGs were all assembled from metagenomes derived from metal and plastic surfaces in New York City. Therefore, *P. septica* clearly appears to be a human-associated species, but insufficient evidence exists to support that it is a pathogen.

### *Pantoea alvi* sp. nov. (76_A_6_B_2_C_1_D_1_E_0_F_)

This species comprises two genomes, the nomenclatural type PSNIH6^Ts^ and UMGS54, with a pairwise ANI of over 99.75%. The corresponding clade is a sister clade of *P. septica*. Strain PSNIH6 was isolated from hospital plumbing, while MAG UMGS54 was assembled from the metagenome of a human gut sample. Therefore, *P. alvi* sp. nov. may occupy a similar ecological niche to its sister species, *P. septica*.

### *Pantoea piersonii* (76_A_6_B_2_C_1_D_0_E_0_F_)

#### Phylogeny, genomics, and reverse ecology

The nine genomes, including that of the type strain DSM 108198^T^, in this species (including three genomes not used in the phylogenetic analysis), have over 96% ANI to each other. The corresponding branch splits off at a node that is basal to the MRCA of *P. septica* and *P. alvi* sp. nov. The species contains two population clusters corresponding to separate clades that are supported in all trees (Supplementary Figures 1–3), one with five members and one with one member. The five genomes of the first cluster share 172 genes that are absent in the genome of the other cluster, while this latter genome has 116 genes absent from the other five genomes, adding further evidence that they represent two distinct populations. Genes unique to either of the two clades include genes encoding phage components, the toxin–antitoxin systems, transporters, multidrug resistance, various enzymes, regulators, antibiotic biosynthesis, and hypothetical proteins (Supplementary Table 4).

#### Ecology, lifestyle, and biotechnological uses

The type strain and some other strains of the species were isolated from environmental samples collected inside the International Space Station, while one strain was isolated from human urine, and three MAGs were sourced from surfaces in New York City. Therefore, bacteria in this species may have an ecological niche associated with humans and may be commonly found in anthropogenic environments without causing disease, similar to bacteria in the related *P. septica* and *P. alvi* species.

### “*Pantoea latae”* (76_A_6_B_2_C_1_D_3_E_0_F_)

This effectively published species (Lata et al., 2017) only has one member, the genome of the type strain AS1^T^, which is located on the branch splitting off at a basal node for *P. piersonii*. Strain AS1^T^ was isolated from the rhizosphere of a Florida Coontie (*Zamia floridana*) plant in Florida, USA (Lata et al., 2017).

### *Pantoea deserta* sp. nov. (76_A_6_B_2_C_1_D_2_E_0_F_)

This species includes the genome of a single strain, the nomenclatural type RIT388^Ts^, isolated from an Ayan tree (*Distemonanthus benthamianus*), known for its antifungal and antibacterial properties (Soutar and Stavrinides, 2019; Gan et al., 2020). The genome is located on the branch splitting off at the most basal node of the clade, which includes *P. septica, P. alvi, P. piersonii*, and *P. latae*.

### *Pantoea* “mediterraneensis” (76_A_6_B_24_C_0_D_0_E_0_F_)

This species consists of the genome of a single strain, the type strain Marseille-P5165^T^, isolated from human skin swabs (Ndiaye et al., 2019; Boxberger et al., 2021). Based on GTDB, previous phylogenomic analyses of the *Erwiniaceae* family (Soutar and Stavrinides, 2019, 2022), and our core genome tree, this species, whose basonym is “*Erwinia mediterraneensis*”, belongs to the genus *Pantoea*. In fact, the genome of Marseille-P5165 shares an MRCA with MAG UBA648^TS^ of *P. superficialis* sp. nov on a branch splitting off at a basal node to all other *Pantoea* genomes but does not cluster with the outgroup *Mixta calida*. We note that, because of their position in the tree, we cannot exclude that *P*. “mediterraneensis” and *P. superficialis* sp. nov. are members of a related genus instead. Additional phylogenetic reconstruction with the genomes of additional genera will need to be performed to confirm their genus affiliation.

### *Pantoea superficialis* sp. nov. (76_A_6_B_24_C_1_D_0_E_0_F_)

This species consists of two MAGs, UBA2655 and the nomenclatural type UBA648^Ts^, both assembled from a metagenome sourced from a metallic or plastic surface in New York City. Only the MAG UBA648^Ts^ was used in the phylogenetic analysis. It shares an MRCA with *P*. “mediterraneensis”.

## Conclusion

By combining phylogenetic core genome reconstruction, genome similarity analysis, a reverse ecology approach, selected gene content comparisons based on a pangenome analysis, and a review of the provenances of samples and the available literature, we have here compiled the description of 49 *Pantoea* species and 25 within-species lineages. After this thorough analysis of 559 genome-sequenced strains, *Pantoea* remains true to its name by being “of all sorts and sources”, with most species and within-species lineages showing little to no obvious specialization to a particular lifestyle or environment.

Only four species include *bona-fide* plant pathogens based on available data at this point (*P. ananatis, P. stewartii, P. allii*, and *P. agglomerans*). Since three of these species (*P. ananatis, P. stewartii*, and *P. allii*) belong to the same larger clade (LINgroup 76_A_6_B_13_C_), the MRCA of this clade may have made the switch to a pathogenic lifestyle. Therefore, any strain that belongs to this clade, even if not a member of the three currently described species, may be more likely to be a plant pathogen than other members of the genus. However, different virulence mechanisms and sporadic plant pathogenicity among some of these species would support caution. Genes specific to this clade were previously identified in a comparative genomics study (Palmer et al., 2018b), where pathogenicity-associated genes included an additional copy of the *rfb* locus for the synthesis of lipopolysaccharides of the O-antigen and a locus encoding aerobactin that may aid in survival in hosts during iron-limiting conditions, among numerous other poorly characterized genes. Although some strains of *P. agglomerans* have been validated as *hrp1* T3SS-dependent gall-forming plant pathogens or pathogens of onion, plant pathogenicity appears to be largely sporadic within the group. Furthermore, other *Pantoea* species include members that were isolated from symptomatic plants; however, Koch's postulates have not been completed, and the sporadic nature of their isolation makes it likely that they live in association with diseased plants but are not the causative agents, such as *P. eucalypti, P. vagans, P. eucrina*, or *P. gossypiicola*.

Three *Pantoea* species, *P. septica, P. piersonii*, and *P. alvi*, appear instead to be mostly human-associated. These three species are also members of a single clade (corresponding to LINgroups 76_A_6_B_2_C_1_D_0_E_ and 76_A_6_B_2_C_1_D_1_E_). Therefore, other strains that belong to this clade may be more likely to have a human/animal-associated lifestyle. There is currently no indication that they are opportunistic human pathogens. Another clade with above-average human-associated isolation sources is the *P. brenneri, P. conspicua, P. astica* sp. nov., and *P*. sp009830035 clade (76_A_6_B_2_C_0_D_1/2/3_E_), whose MRCA may thus also have adapted to a more human/animal-associated niche. However, for this clade, there is no indication of a pathogenic lifestyle. With future increased genomic representation of these groups, further comparative genomics analyses may shed light on the adaptations associated with shifts in lifestyles.

All other species with more than one or two members appear to be associated with multiple sources, mainly plants, soil, insects, fungi, air, and water. Furthermore, the distinct within-species lineages, whose circumscription is supported by phylogenomics, recent HGT events, and differences in gene content, still seem to be generalists. Therefore, there appears to be little adaptation and specialization to different ecological niches in most species of the genus, even when a within-species lineage becomes evolutionarily isolated. Bacteria may instead be frequently migrating between environments, for example, from plants to the water cycle and/or soil and back to plants, as suggested by the finding that rain-borne *Pantoea* bacteria are efficient colonizers of plant leaves (Mechan Llontop et al., 2021).

*P. agglomerans* deserves particular attention since it is considered to be a biosecurity risk based on reports indicating a potential for causing human infections (Bonaterra et al., 2014). However, only three out of over 120 genome-sequenced members of *P. agglomerans* were isolated from human tissues or wounds, and it is unclear if they were causative agents of infection. Therefore, based on the data analyzed here, we are not able to further support or reject an elevated biosecurity risk for *P. agglomerans* overall or for any of its three within-species lineages.

In general, a lack in the clear correlation of isolation sources with taxonomic assignment and a lack of evidence for pathogenicity based on currently available data of genome-sequenced isolates indicate lower pathogenic potential for several species in the genus compared to what one would expect based on literature. For example, several recent reviews report the pathogenicity of some *Pantoea* species to plants and/or humans (Bartlett et al., 2022; Yang et al., 2023). However, when one looks at the evidence, in several cases, either Koch's postulates were not completed and/or bacteria were only identified based on partial 16S rRNA sequencing, which is insufficient for the identification at the species rank, particularly in the *Enterobacteriaceae*.

Another problem in assessing the biosecurity risk of *Pantoea* species is the misidentification or mislabeling of genes and genomes in NCBI databases. GTDB (Parks et al., 2022) has significantly reduced this confusion by assigning genomes to a solid genome-based taxonomy. However, GTDB does not provide descriptions of species, and many species clusters only have provisional names. Furthermore, being a taxonomy database, phylogenetic information between the genus rank and species rank and within species is not provided. By reconstructing phylogenetic relationships from the rank of genus to the level of genetic lineages within species, naming new species using the SeqCode (Hedlund et al., 2022), and delineating and describing these lineages, we have further improved the taxonomy of the genus *Pantoea*. Note that naming under SeqCode was not possible for some species clusters because raw sequencing data were not available for any of the member genomes, and, therefore, none of the genomes could be used as a nomenclatural type for those species.

Importantly, any newly sequenced genomes in the *Pantoea* genus that will be added to LINbase in future will be automatically classified based on ANI, including the genomes that have been added to GTDB as part of release 214 after the analysis for this study was completed. Even if these genomes do not belong to any of the species and lineages delineated here or any newly named species, it would still be possible to identify them since the LINs assigned to these new genomes will precisely express their similarity to all previously sequenced *Pantoea* genomes. The caveat is that, since we did not observe the specialization of lineages to specific lifestyles, most importantly, pathogenicity to plants or animals, such precise identification, may still not allow the prediction of any relevant phenotypes.

In conclusion, our analysis establishes a solid genome-based framework for the precise identification of *Pantoea* isolates. As a next step, already available isolates will need to be thoroughly phenotyped with regard to their pathogenicity to plants and animals to determine the biosecurity risk they present; genome comparisons need to be extended to the analysis of confirmed or putative virulence genes; and sampling needs to continue to further our knowledge of the phenotypic and genotypic diversity that exists within the genus (note that since the writing of this study and completion of genome analyses, new genomes, and species clusters have already been added to GTDB Release 214, and we will update LINbase with these genomes and species clusters). Making progress in regard to all three of these aspects will lead us toward a more meaningful classification and identification of the genus, which will allow for better protection of human, animal, and plant health from pathogenic members, while expanding the deployment of beneficial members in biotechnology and biocontrol.

## Data availability statement

The datasets presented in this study can be found in online repositories. The names of the repository/repositories and accession number(s) can be found below: https://www.ncbi.nlm.nih.gov/, PRJNA445714.

## Author contributions

KC: Data curation, Investigation, Writing—original draft, Writing—review & editing. MR: Data curation, Formal analysis, Investigation, Methodology, Validation, Visualization, Writing—review & editing. PS: Investigation, Methodology, Supervision, Writing—review & editing. MJ: Investigation, Methodology, Supervision, Writing—review & editing. RM: Data curation, Investigation, Software, Writing—review & editing. BK: Validation, Writing—original draft, Writing—review & editing. TS: Validation, Writing—original draft, Writing—review & editing. SV: Writing—original draft, Writing—review & editing. TC: Writing—original draft, Writing—review & editing. LSH: Conceptualization, Writing—review & editing. MP: Data curation, Validation, Visualization, Writing—original draft, Writing—review & editing. BAV: Conceptualization, Data curation, Funding acquisition, Investigation, Methodology, Project Writing—review & editing.

## Supplementary material

Supplementary Figure 1 Core genome phylogenetic tree prepared with IQ-TREE. Phylogeny shown in Figure 1 with strain names included together with population cluster assignment (in brackets next to strain names). In addition to clades corresponding to species, subclades within species are also annotated. Branch support is inferred with ultrafast bootstrapping from 1,000 replicates. The same color scheme to Figure 1 is used to denote species. Type strains and type sequences are followed by a “T” or “Ts”, respectively.

Supplementary Figure 2 Core genome phylogenetic tree prepared with FastTree. Approximate ML phylogenetic tree of the genus *Pantoea* of the same genomes used in Supplementary Figure 1. Shimodaira-Hasegawa-like branch support values from 1,000 replicates are indicated as colored dots at nodes.

Supplementary Figure 3 Pangenome phylogenetic tree made with IQ-Tree. ML phylogenetic pangenome tree of *Pantoea* constructed with IQ-TREE. Branch support was inferred with ultrafast bootstrapping from 1,000 replicates and are indicated as colored dots at the nodes.

Supplementary Table 1 Metadata file. Information on the strains and genomes within the genus *Pantoea*, including phylogenetic tree assignment, Life Identification Number (LIN) assignment, species names, National Center for Biotechnology Information (NCBI) metadata, including taxonomic lineage information, Genome Taxonomy Database (GTDB) metadata, including taxonomic lineage information, CheckM results, and genomic information (Parks et al., 2015; Hugenholtz et al., 2021).

Supplementary Table 2 Distribution of isolation sources for each *Pantoea* species. Information on the species within the genus *Pantoea*, including genomic information from the National Center of Biotechnology (NCBI) and general information from the List of Prokaryotic Standing in Nomenclature (LPSN) and Genome Taxonomy Database (GTDB) (Parte et al., 2020; Hugenholtz et al., 2021).

Supplementary Table 3 Species and lineages circumscribed as LINgroups in LINbase. This table includes LINs of *Pantoea* species, lineages above species rank, and within-species lineages.

Supplementary Table 4 Gene differences between selected within-species subclades.
