# Supplementary material for: Genomic delineation and description of species and within-species lineages in the genus Pantoea
Source: Front Microbiol. 2023 Nov 9;14:1254999. doi: 10.3389/fmicb.2023.1254999 (PMC10665919; doi:10.3389/fmicb.2023.1254999)
Supplement: Supplementary Figure 1 — Core genome phylogenetic tree prepared with IQ-TREE. Phylogeny shown in Figure 1 with strain names included together with population cluster assignment (in brackets next to strain names). In addition to clades corresponding to species, subclades within species are also annotated. Branch support is inferred with ultrafast bootstrapping from 1,000 replicates. The same color scheme to Figure 1 is used to denote species. Type strains and type sequences are followed by a “T” or “Ts”, respectively. [file Image_1.pdf]

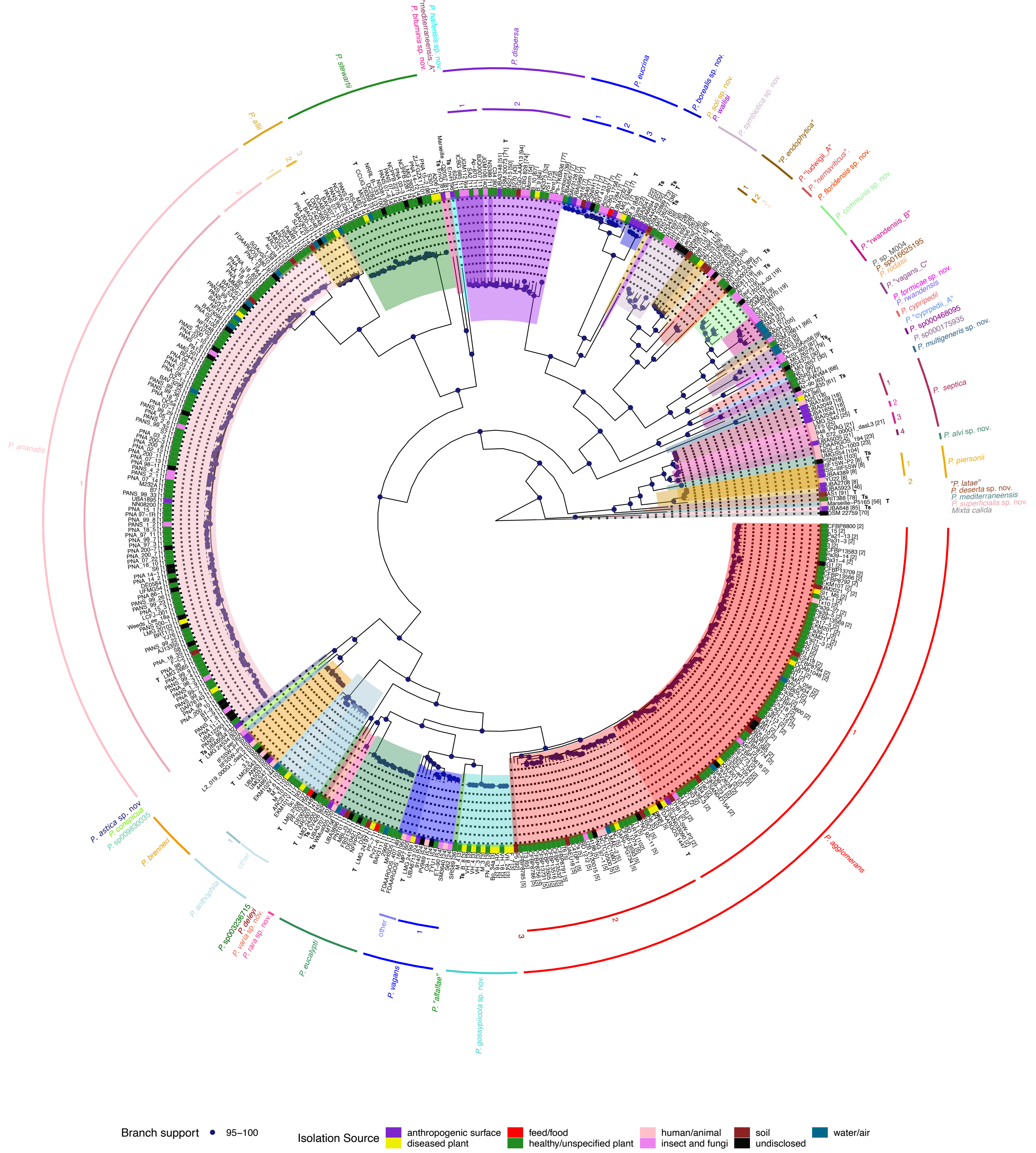
P. stewartii
P. "mediterraneensis_A"
P. haifensis sp. nov.
P. bituminis sp. nov.
P. dispersa
P. eucrina
P. borealis sp. nov.
P. soli sp. nov.
P. wallisii
P. symbiotica sp. nov.
"P. endophytica"
P. "ludwigii_A"
P. "nemavictus"
P. floridensis sp. nov.
P. communis sp. nov.
P. "rwandensis_B"
P. sp. M004
P. sp016625195
P. rodasii
P. "vagans_C"
P. formicae sp. nov.
P. rwandensis
P. cypripedii
P. "cypripedii_A"
P. sp000468095
P. sp000175935
P. multigeneris sp. nov.
P. septica
P. alvi sp. nov.
P. piersonii
"P. latae"
P. deserta sp. nov.
P. mediterraneensis
P. superficialis sp. nov.
Mixta calida
P. agglomerans
P. gossypiicola sp. nov.
P. "alfalfae"
P. vagans
P. eucalypti
P. sp003236715
P. deleyi
P. varia sp. nov.
P. rara sp. nov.
P. anthophila
P. brenneri
P. astica sp. nov.
P. conspicua
P. sp009830035
P. ananatis
P. allii
Branch support
95–100
Isolation Source
anthropogenic surface
diseased plant
feed/food
healthy/unspecified plant
human/animal
insect and fungi
soil
undisclosed
water/air

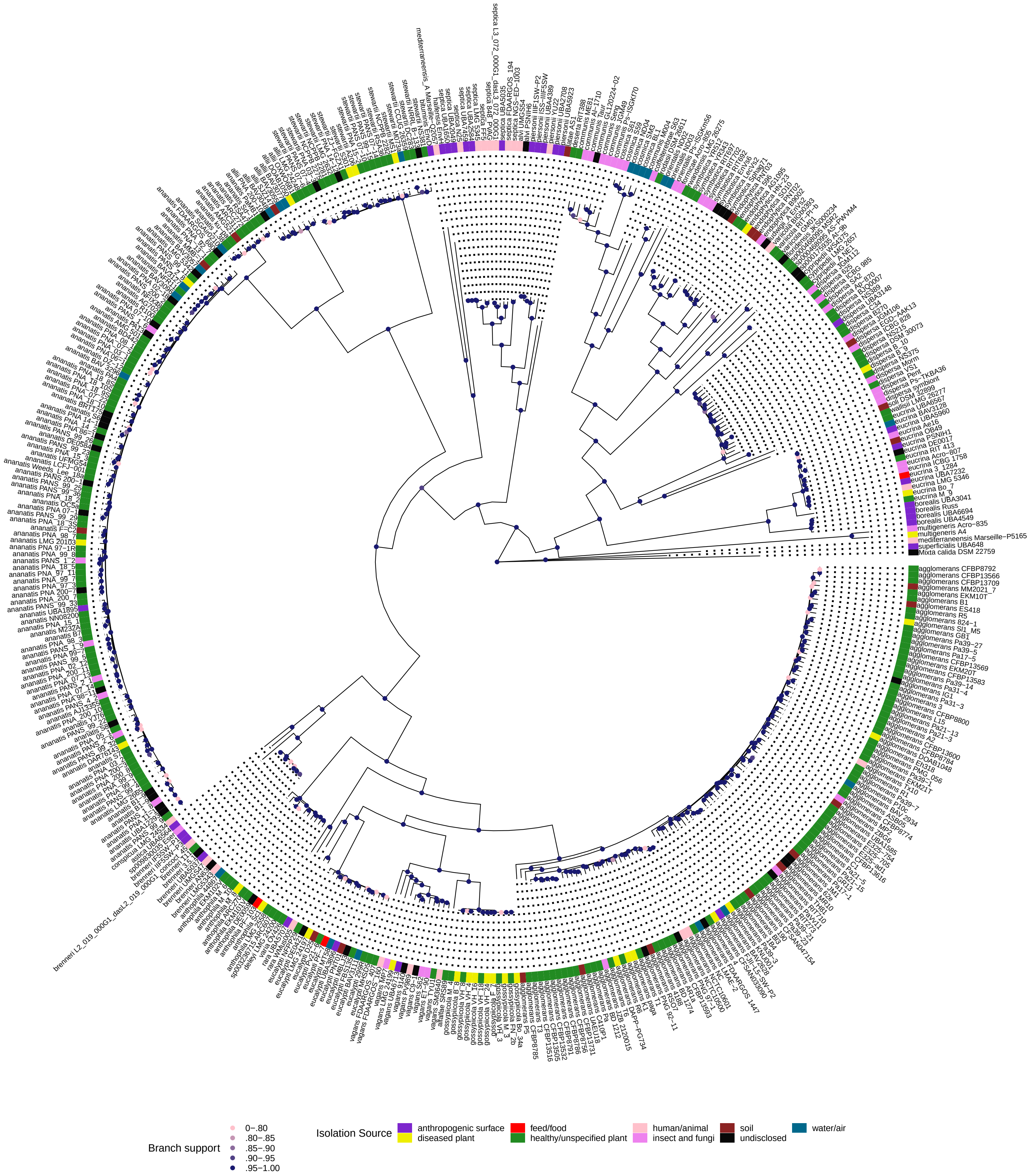

Branch support
0–.80
.80–.85
.85–.90
.90–.95
.95–1.00
Isolation Source
anthropogenic surface
diseased plant
feed/food
healthy/unspecified plant
human/animal
insect and fungi
soil
undisclosed
water/air

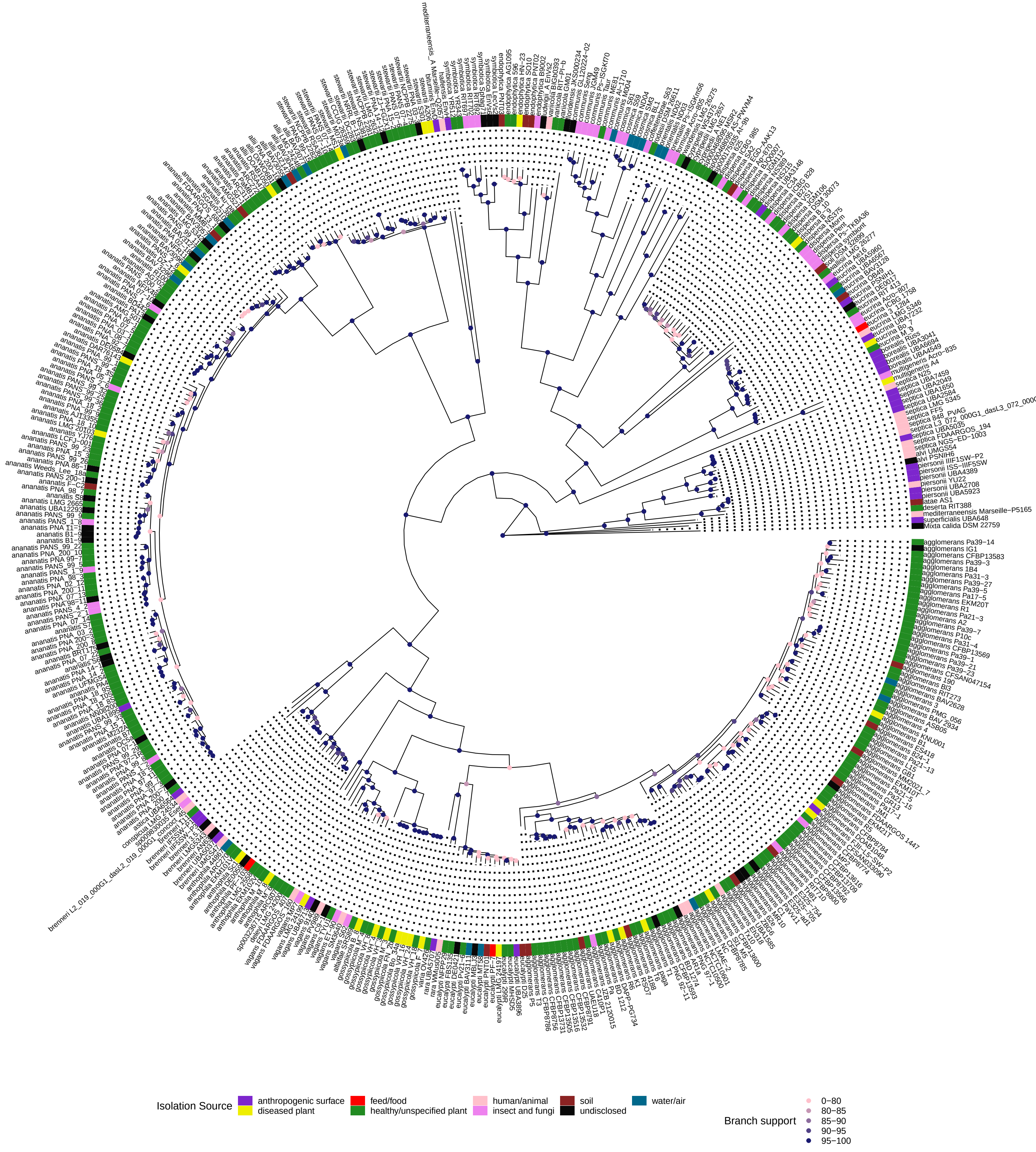
Isolation Source
anthropogenic surface
diseased plant
feed/food
healthy/unspecified plant
human/animal
insect and fungi
soil
undisclosed
water/air
Branch support
0–80
80–85
85–90
90–95
95–100
